# Advanced Nanostructured Coatings Based on Doped TiO_2_ for Various Applications

**DOI:** 10.3390/molecules28237828

**Published:** 2023-11-28

**Authors:** Mariuca Gartner, Anna Szekeres, Hermine Stroescu, Daiana Mitrea, Maria Covei

**Affiliations:** 1 Institute of Physical Chemistry “Ilie Murgulescu”, Romanian Academy, 202 Splaiul Independentei, 060021 Bucharest, Romania; 2 Institute of Solid State Physics, Bulgarian Academy of Sciences, Tzarigradsko Chaussee 72, 1784 Sofia, Bulgaria; 3 Department of Product Design, Mechatronics and Environment, Transilvania University of Brasov, 29 Eroilor Bd., 500036 Brasov, Romania

**Keywords:** titanium dioxide, structure and properties, photocatalytic applications, gas sensors and biosensors

## Abstract

For many years, TiO_2_-based materials and improving their properties in order to expand their application areas have been the focus of numerous research groups. Various innovative approaches have been proposed to improve the photocatalytic and gas-sensing properties of TiO_2_ nanostructures. In this review, we aim to synthesize the available information in the literature, paying special attention to the sol–gel technology, which is one of the most frequently used methods for TiO_2_ synthesis. The influence of dopants on the structural, morphological, optical, and electrical properties of TiO_2_ and the way to modify them in a controlled manner are briefly discussed. The role of shallow and/or deep energy levels within the TiO_2_ bandgap in the electron transport behavior of doped TiO_2_ is emphasized. Selected research on photocatalytic applications in water disinfection, wastewater treatment, and self-sterilizing coatings that contribute to improving the quality of human life and environmental preservation is highlighted. A survey of biosensors that are closely related to medical applications such as cancer detection, implantology, and osteogenesis is also provided. Finally, the pressing problems that need to be solved in view of the future development of TiO_2_-based nanostructures are listed.

## 1. Introduction

Titanium dioxide (TiO_2_) is one of the most popular commercially available nanomaterials, with various application fields due to its low cost, lack of toxicity, wide availability, biocompatibility, and high chemical stability. TiO_2_ has also received extensive attention because of its strong photocatalytic activity, which has been the subject of numerous research studies.

**Figure 1:**
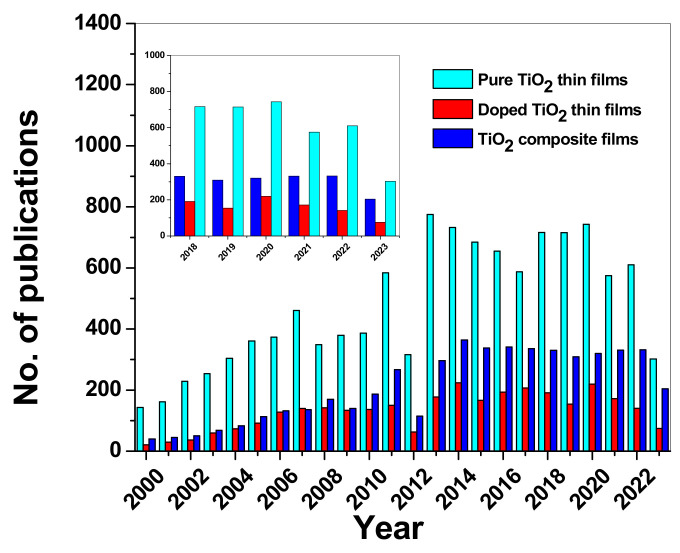
Comparative number of publications about pure, doped, and composite TiO_2_ thin films published between 2000 and 2023. Inset: illustration of the last five years. Source: Scopus (accessed on 25 September 2023).

In the past five years, there have been a significant number of publications focused on exploring the applications of TiO_2_. The comparative evolution of the paper numbers in these years is presented in Figure 1.

As a wide energy band gap material, TiO_2_ can be activated only under UV irradiation, which constitutes up to 10% of the solar spectrum. Therefore, several methods have been researched to overcome this shortcoming of TiO_2_ photocatalysis by shifting and enhancing the absorption toward the visible region. Improving the properties of TiO_2_-based materials can be achieved by the following pathways: adjustment of synthesis parameters (concentration, pH, temperature, reaction time, stirring time, and annealing treatment), elemental doping (metallic or non-metallic), bandgap engineering, construction of heterojunctions, and surface modification by treatment with inorganic acids.

Doping with metal atoms and combining with 2D nanomaterials such as graphene, (reduced) graphene oxide, or carbon nitride are some of the more recent attempts to enhance the photocatalytic properties of TiO_2_. The synthesis of a 2D TiO_2_ hybrid nanomaterial resulted in a decrease in the band gap energy and a delay in the recombination of photo-generated electron–hole pairs during the photocatalytic process. Moreover, it was shown that the higher surface area of the 2D composite promotes better adsorption of the pollutant molecules to the photocatalyst surface during photocatalysis [1].

The photocatalytic activity of the TiO_2_ material was intensively exploited for self-cleaning, food storage, wastewater treatment, air purification applications, etc. Furthermore, TiO_2_ was used as a white pigment to develop paints or washable paints, printing inks, and also as a UV filter in cosmetics.

In contrast, the application of TiO_2_ for gas sensing has revolutionized sensor technology in recent years. TiO_2_ sensors have proven to be invaluable in numerous applications, providing precise and reliable data in various industries. Numerous experiments have been conducted to improve the sensitivity of TiO_2_. The construction of multi-component nano heterostructures in gas sensing is a promising solution that shows superior sensing performance over that of single-component sensors.

Although, due to its properties, TiO_2_ can be used in various fields, it is important to mention that there are also some risks and limitations to the material. For example, in the field of wastewater treatment, the most efficient photocatalyst is most often in nano powder form, which can be difficult and costly to completely recover from the water stream. The International Agency for Research on Cancer has classified TiO_2_ nanoparticles as posing potential risks to human health. They are particularly dangerous if inhaled either during the production or the use of lifetime stages. This is why, for example, a recent European directive has banned the use of TiO_2_ in food dyes [2]. However, if TiO_2_ nanoparticles are ingested at low concentrations, they do not pose a significant threat to human health [3].

A review on doped TiO_2_ was written in 2008 by A. Zaleska [4], in which a comprehensive table of metal and non-metal dopants for TiO_2_ in photocatalytic applications was presented. One of the challenges with doped TiO_2_ remains the loss of photoactivity over time at the end-of-life stages of the material. Previously, the efficiency of the metal-doped TiO_2_ under visible light was linked to the preparation method used. A list of patents for metal- and non-metal-doped TiO_2_ was discussed too.

The most recent reviews on TiO_2_ are presented in Table 1.

The purpose of the present work is to highlight the unique properties and most important uses of TiO_2_-based materials as photocatalysts and biosensors and to provide updated information regarding this topic. To this end, a brief overview of the intrinsic properties of TiO_2_ is essential, along with a discussion of the structural, morphological, optical, and electrical properties of both pure and doped TiO_2_ films. Special attention is given to the sol–gel (SG) technology, as it is one of the most frequently used methods of TiO_2_ synthesis.

## 2. Properties of TiO_2_ Films

In this section, we will briefly consider the structure, optical, and electronic properties of TiO_2_ material and the possibility to modify them in a controlled manner to meet the requirements of specific applications. As mentioned above, TiO_2_ has unique properties that make it very useful in many industrial branches as a material in electrochromic, photovoltaic, and microelectronic devices, gas sensors, photocatalysts, coatings, implants, etc. The effective use of TiO_2_ for these applications largely depends on its structure (polymorph form, shape, and arrangement of nanocrystallites) and defects (intrinsic defects and extrinsic impurities), which in turn strongly depend on the preparation technologies. Our attention is focused on how dopants (metal or non-metal atoms) affect the properties of TiO_2_ films.

### 2.1. Structural Properties

Among the various crystal phases of titania, the anatase (tetragonal), rutile (tetragonal), and brookite (orthorhombic) phases are of greater importance for different applications. Depending on thermal treatment methods, the phase transition of TiO_2_ occurs in the order of amorphous to anatase (375 °C), brookite (510 °C), and rutile (650 °C). In the tetragonal crystal structure of anatase (a = b = 0.378 nm, c = 0.95 nm, space group I4_1_/amd) and rutile (a = b = 0.4593 nm, c = 0.2959 nm, space group P4_2_/mnm), the titanium atom is surrounded by six oxygen atoms, and each oxygen atom is surrounded by three titanium atoms [15,16]. Each octahedron shares corners, leading to the formation of (001) planes. Each crystalline phase offers distinctive characteristics that make it suitable for different applications: rutile is preferred for its thermal stability and optical properties, anatase for photocatalytic and surface-area-related applications, while brookite has more limited use due to its relative scarcity and lower stability. From the point of view of gas sensor applications, the rutile and anatase TiO_2_ crystal forms are of interest and are most intensively investigated. Generated by light illumination of electron–hole pairs in anatase, TiO_2_ can initiate various photocatalytic reactions, such as the degradation of organic pollutants.

**Figure 2:**
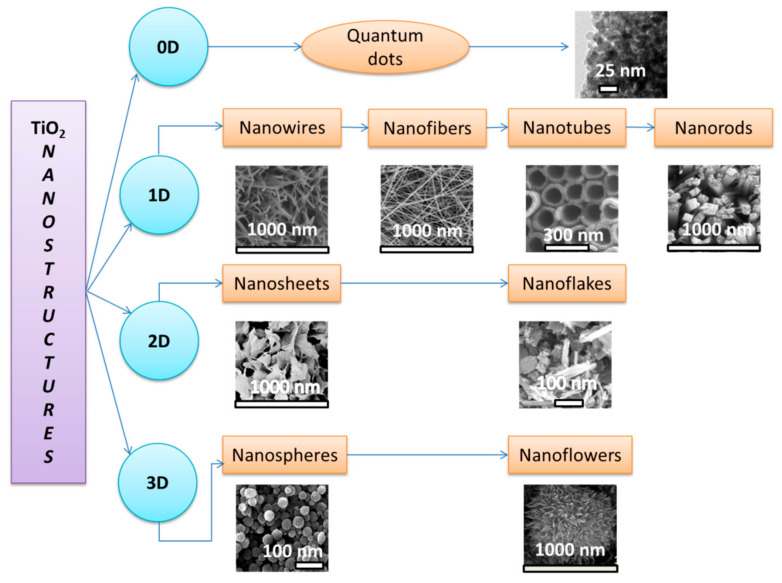
TiO_2_ nanostructures: 0D, 1D, 2D, and 3D. Reprinted from [17,18,19,20,21,23,24,25] with permission from Elsevier. Reprinted with permission from [22]. Copyright © 2018 American Chemical Society.

With the increasing demand for nanostructured materials, extensive research has been conducted to develop technologies to obtain nanocrystalline TiO_2_ structures that would meet the requirements of given applications. It was found that a reduction in particle size improves various TiO_2_ properties, revealing the superior performance of nanocrystallites. In Figure 2, the dimensional diversity of TiO_2_ nanostructures is illustrated, starting from zero-dimensional nanoparticles (0D) and reaching hierarchical or complex TiO_2_ nanoparticle structures (3D), which are very important in choosing between the numerous applications of TiO_2_.

By controlling the synthesis parameters, especially pH, temperature, and thermal annealing, various morphologies with improved properties can be achieved. Accordingly, different nanostructures were prepared using synthesis methods [17,18,19,20,21,22,23,24,25] such as the following: classical (for nanorods, nanosheets, nanoflakes, and nanoflowers) or ultrasonic-assisted hydrothermal method (for quantum dots), electrospinning (for nanofibers), dip coating and hydrothermal routes (for nanowires), anodization (for nanotubes), and template-assisted methods (for nanospheres). Using the aforementioned hierarchical structures, an improvement to the different parameters was observed, e.g., an increase in the active surface area, a better charge carrier separation with a smaller recombination rate, etc.

The incorporation of dopants into the TiO_2_ lattice has a strong impact on the structural properties of TiO_2_, such as promoting phase transitions and/or changing lattice parameters and generating defects that are activated by external influences (electric field, radiation, etc.).

The growth of specific crystal phases, such as anatase, rutile, or brookite, can be stimulated by certain dopants and, in turn, has a significant effect on the material’s properties. Kondamaredd et al. [26] investigated the effect of doping using tungsten ions (W^6+^) on a nano-crystalline structure of pristine anatase TiO_2_ by varying the ion concentration (10, 50, 90, and 120 ppm) using the sol–gel and hydrothermal methods. X-ray diffraction (XRD) analysis pointed out the phase transition from anatase to rutile, which was more pronounced when the concentration of W increased up to 50 ppm. Additionally, certain dopants can alter the phase transition process and thermal stability. For example, certain dopants can slow down phase transitions between anatase and rutile phases, leading to materials with improved phase stability at higher temperatures. Zhu et al. [27] studied the process of phase transition from the anatase form to the rutile form in TiO_2_ doped with 5 and 7.5% Si by in situ high-temperature XRD. They observed an increase in the phase transition temperature and activation energy while increasing the dopant percentage. This result showed that by introducing Si into the TiO_2_ lattice, the phase transition of TiO_2_ from anatase to rutile is inhibited, while the crystal-growth process is controlled by the crystal interface growth. In a recent study, Zhang et al. [28] showed that doping TiO_2_ with Fe atoms leads to an increase in the temperature of the phase transition from anatase to rutile, while a reduction in Fe^3+^ to Fe^2+^ generates oxygen vacancies upon Fe doping, accelerating the phase transition process.

In general, the dopants have different atomic sizes compared to the host atoms, leading to lattice expansion or contraction. Lattice distortion can affect the mechanical properties of the deposited films as well as their electronic structure. For instance, Kayani et al. [29] synthesized V-doped TiO_2_ thin films with different percentages of V doping (1, 3, 5, 7, and 9 at. wt. %) using the sol–gel technique (the dip-coating method). The incorporation of V ions created defects and distortion in the TiO_2_ lattice. The increase in the dopant percentage led to a reduction in the degree of crystallinity and crystallite size, and consequently, a decrease in the specific surface area. These modifications are related to the ionic radius of V^5+^ (0.059 nm) being lower than that of Ti^4+^ (0.062 nm). An introduction of dopants in the oxide network induces lattice defects such as vacancies, interstitials, and impurities. Although these defects do not cause structural changes, they will create localized energy levels in the oxide band gap, affecting electronic and optical properties, as we will discuss later. For example, Park et al. [30] studied nitrogen-doped TiO_2_ heterostructures prepared by graft polymerization. The FT-IR spectra showed several absorption bands related to the vibration of N-H, CO, and CN bonds upon polymerization of the grafted poly(methacrylamide) (PMAAm) on TiO_2_ nanoparticles. With the appropriate thermal treatment, the PMAAm molecules/particles were completely removed, and a uniform N-doped TiO_2_ with a significantly narrowed band gap was formed. The newly formed N 2p band above the O 2p valence band induced a significant narrowing of the TiO_2_ band and caused a red shift of the absorption edge in the visible region. Compared to pure TiO_2_, these N-doped TiO_2_ heterostructures exhibited improved photocatalytic performance when exposed to visible light, which was attributed to a modification in the electronic band structure of TiO_2_.

As shown above, the influence of dopants on their structural properties depends on their size, charge, concentration, interaction with the host lattice, etc. By selecting and controlling the type and concentration of dopants, new structural characteristics are developed that can be used for specific applications, ranging from catalysis, sensors, electronics, and energy conversion to medicine and healthcare.

### 2.2. Morphological Properties

Nowadays, most of the TiO_2_ films that have found practical applications have a nanocrystalline structure. Therefore, it is extremely important to understand how (intrinsic and extrinsic) defects affect the morphological properties in order to design TiO_2_ materials with the desired characteristics for specific applications. The doping of TiO_2_ films again has a decisive influence on many processes, such as dopant incorporation, crystal growth kinetics, and surface interactions.

**Figure 3:**
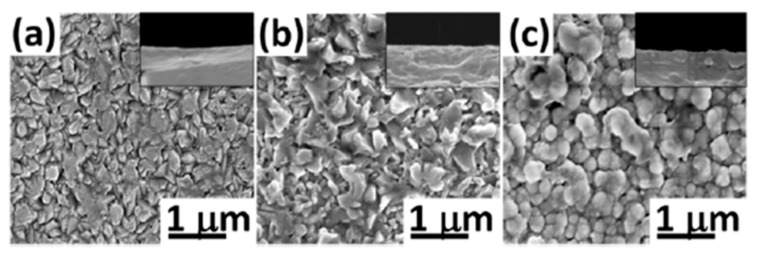
SEM images of typical (**a**) undoped TiO_2_ (**b**) low-concentration, and (**c**) high-concentration P-doped TiO_2_ films. The inset shows a side-view SEM of the films. Reprinted with permission from [32]. Copyright © 2015 American Chemical Society.

The nucleation and growth kinetics of TiO_2_ nanocrystallites can be altered using dopants. Depending on the type and concentration of dopants, as well as the technology used to deposit doped TiO_2_, the grown particles will have different sizes and shapes, as the dopants can promote the formation of specific crystal facets, leading to modified particle shapes. For example, in Elmehasseb et al. [31], the effect of doping with N, S, and Zn of sol–gel TiO_2_ films is expressed by a decrease in the particle sizes, which become smaller (42–70 nm) and less agglomerated compared to those (61–89 nm) observed for pure TiO_2_. The opposite effect was observed in phosphorus-doped TiO_2_ films prepared by APCVD at 473 K [32]. The incorporation of P in the film structure caused a drastic change in the structural morphology and increased the electrical conductivity. Scanning electron microscopy (SEM) images (Figure 3) visualized the enlarging nanoparticle sizes in the anatase TiO_2_ structure while increasing the P^5+^ species in the films. This study showed that it is possible to obtain novel multifunctional materials with an optimal balance between self-cleaning and TCO properties as photocatalytic transparent conductors.

Recently, Asrafuzzaman et al. [33] employed an interesting biogenic method to obtain pure and doped TiO_2_ nanoparticles from mango leaves. They introduced Cu and Ag transition metals as dopants in concentrations ranging from 0.5% to 2%. A morphological analysis revealed that undoped TiO_2_ particles had a spherical shape, while both Ag-doped and Cu-doped samples exhibited particle agglomeration. Although the photocatalytic efficiency of doped TiO_2_ was found to be higher than that of undoped TiO_2_, minimizing the agglomeration of TiO_2_ nanoparticles is crucial to further improving the photocatalytic performance.

In some cases, dopants can also influence the growth dynamics of TiO_2_ crystals. For example, Avilés-García et al. [34] studied co-doping with Mo and W of TiO_2_ through the evaporation-induced self-assembly (EISA) method. The obtained co-doped TiO_2_ had smaller crystallite sizes and higher crystallinity than TiO_2_ with only one dopant.

Dopants can also promote the formation of nanostructures in TiO_2_ materials, such as nanowires, nanotubes, and nanosheets. These nanostructures have unique morphological properties that are useful for specific applications such as nanoelectronics and energy storage. Several related studies have been reported considering the changes in the morphology of TiO_2_ structures caused by various factors, such as the technological conditions of different film preparation methods, the type and concentration of the dopants, etc. [32,33,34,35,36,37,38,39].

It is known that dopants are able to influence the surface roughness of doped films. Recently, Bhandarkar et al. [37] studied the effect of the Mn dopant on the properties in TiO_2_ films. The AFM image of the undoped samples revealed densification when the small crystallites merged together. Analyzing the AFM images, it was shown that the surface roughness values increase with the Mn concentration. Specifically, the undoped TiO_2_ thin films exhibit the lowest value at 3.2 nm, which rises to 4.1 nm for the sample doped with 8 at.% Mn, which could be attributed to the merging of smaller crystallites.

### 2.3. Optical Properties

Optical properties depend on how light propagates in the solid and how much of that light is absorbed in the material. This process depends on the dielectric permittivity of the material, which in turn is determined by the structure of the energy bandgap and the free electrons of TiO_2_. All the TiO_2_ polymorphs have high relative permittivity (ε_ox_ > 30) and refractive index (~1.93 < n < 2.6 at λ = 633 nm), high transparency in the visible spectral range (~80% transmittance), and a wide optical bandgap (E_g_ > 3 eV). In general, metal oxides with an energy band gap larger than 3 eV have no absorption in the visible range of light. Therefore, TiO_2_ is a promising candidate among dielectrics as a high refractive index material and transparent coating in multilayer optical systems. However, to exploit the full capacity of Titania in other areas of applications, TiO_2_ films must be doped with appropriate metal or non-metal atoms. Dopants affect the optical properties of TiO_2_ by introducing energy levels within the bandgap, which allows visible light to be absorbed and thus changes the oxide’s transparency.

The band gap energy of high-quality pure TiO_2_ is significantly large and depends on its crystalline phase. Amorphous TiO_2_ has the widest bandgap, while in crystalline form, the anatase phase has the largest band gap compared with the phases of rutile and brookite, accordingly, E_g_ ~ 3 eV (rutile) < E_g_ (brookite) < E_g_ ~ 3.2 (anatase)< E_g_ = 3.5 eV (amorphous) [15,17]. The bandgap energy value of each crystalline phase varies with the preparation method and the presence and concentration of the intrinsic/extrinsic defects. From these, it follows that by controlling the TiO_2_ band gap energy, the optical properties can be engineered.

Over the last decade, a significant number of publications have appeared where different deposition methods have been applied to develop appropriate TiO_2_ structures for given applications, most of which investigated the role of dopants in controlling the optical properties of the prepared structures [32,38,40,41,42,43,44,45,46,47].

Recently, Lettieri et al. [48] gave a very comprehensive review of the basic knowledge on the charge carrier processes that determine the optical and photophysical properties of intrinsic TiO_2_, surveying 315 articles on the related research topic. They discuss in detail the elementary photocatalytic processes in an aqueous solution, including the photogeneration of reactive oxygen species (ROS) and the hydrogen evolution reaction for hydrogen (H_2_) production. In particular, the authors outline the strategies based on highly reduced TiO_2_ (referred to as “black TiO_2_”), as well as facet-engineered nanocrystals and heterojunction photocatalysts, where TiO_2_ is electronically coupled with a different material acting as a co-catalyst.

**Figure 4:**
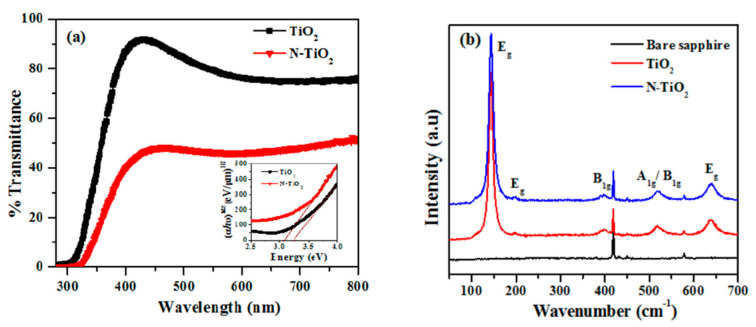
(**a**) Optical transmittance spectra and (**b**) Raman spectra of epitaxial anatase TiO_2_ and N-doped TiO_2_ thin films on Al_2_O_3_ substrate. Reprinted with permission from [41]. Copyright © 2016 American Chemical Society.

Extending the light absorption range of TiO_2_ into the visible region can be achieved by creating localized energy levels within the bandgap, which facilitates the excitation of electrons from the valence band to the dopant-induced energy levels, increasing the material’s light absorption capacity. In [41], the effect of N doping on the structural, optical, electrical, and magnetic properties of epitaxial anatase TiO_2_ films prepared by the atomic layer deposition (ALD) method has been studied. N doping lowered the bandgap value of 3.23 eV for the undoped film to 3.07 eV (Figure 4a), which was attributed to the generation of titanium vacancies by N dopants in an anatase oxide lattice (Figure 4b), enhancing *p*-type conductivity and amplifying room-temperature ferromagnetism in these films.

It has been observed that the V dopant introduced into the TiO_2_ structure (E_g_ = 3.08 eV) lowers the bandgap energy to 2.22 eV for TiO_2_:V and decreases the recombination rate [42]. These TiO_2_:V structures could be used in applications targeting the visible region of light, such as the successful photodegradation of the Acid Yellow 36 (AY36) dye from textile wastewater. In [43], the doping effect of nitrogen on TiO_2_ nanotube arrays (TiO_2_ NTAs) was observed as a bandgap narrowing from 3.16 eV (undoped TiO_2_ NTAs) to 2.9 and 2.7 eV for N-doped and self-doped TiO_2_. On the other hand, co-doping was also performed for the same purpose [44], using Mn^2+^ and Co^2+^ dopants (Mn-Co-TiO_2_) for the photocatalytic degradation of enoxacin (ENX) under solar light irradiation. In this case, the calculated E_g_ values for TiO_2_, Mn-TiO_2_, Co-TiO_2_, and Mn-Co-TiO_2_ were 2.81, 2.62, 2.50, and 2.10 eV, respectively, as the lower E_g_ values are for the doped samples [44].

**Figure 5:**
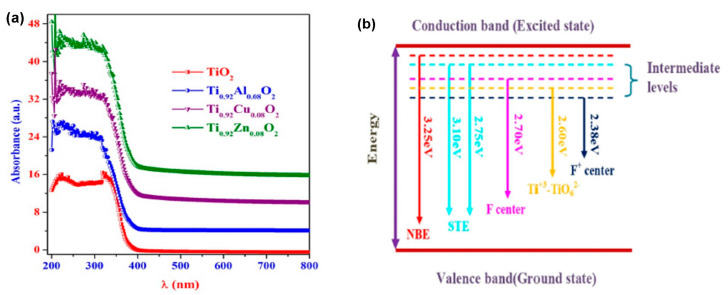
(**a**) UV–Vis–NIR absorption spectra and (**b**) band diagram illustrating possible PL emissions (electronic transition) in undoped and doped Al^+3^, Cu^+2^, and Zn^+2^ TiO_2_ NPs. Reprinted from [45] with permission from Elsevier.

Another example of how the structure and optical properties of TiO_2_ can be modified is given in [45], where undoped and doped Al^+3^, Cu^+2^, and Zn^+2^ (8 at.% each) TiO_2_ NPs prepared by green sol–gel synthesis have been studied. TEM micrographs showed high crystallinity and a narrow size distribution of anatase nanocrystallites (3–8 nm) in the undoped and doped samples. UV–Vis–NIR absorption spectra registered a red shift in the absorption edge for doped TiO_2_ NPs (Figure 5a). It has been established that the incorporation of Al^+3^, Cu^+2^, and Zn^+2^ atoms in the TiO_2_ NPs lattice leads to lattice distortion and generated F^+^ defect centers and oxygen vacancies, which create intra-band states in the energy band gap and cause the observed reduction in the band gap values (Figure 5b).

The above results revealed the specific influence of dopants on the energy band gap of TiO_2_, varying the dopant’s energy levels, concentration, and synthesis conditions. By selecting and controlling the desired dopants, the absorption edge can be tuned, and thus, the optical properties of TiO_2_ films can be tailored for a large range of applications, from photocatalysis and solar cells to sensors, light-emitting devices, etc.

### 2.4. Electrical Properties

Pure and stoichiometric TiO_2_ is an insulator at both room and moderate temperatures with an extremely high specific resistivity in the order of 10^8^ Ωcm. It is a wide bandgap semiconductor, and its bandgap energy depends on TiO_2_ crystalline phases (see Section 2.3). In stoichiometric TiO_2_, the almost complete absence of free carriers results in a full valence band and an empty conduction band.

The common feature of TiO_2_ films prepared by various technological methods is that the obtained films are no longer stoichiometric, as they have a complex defect structure and an increased number of intrinsic defects. These defects can be oxygen vacancies (V_O_), titanium interstitials (Ti_int_), titanium vacancies (V_Ti_), or oxygen interstitials (O_int_). The predominant defects are oxygen vacancies (V_O_) and titanium interstitials (Ti_int_), and both are n-type defects, creating shallow donor states below the conduction band in the TiO_2_ energy gap. This explains why pure TiO_2_ is a native n-type semiconductor.

Depending on whether the film is oxygen- or titanium-deficient, it appears as an amphoteric semiconductor and exhibits an n-p transition as an intrinsic property [49,50,51]. This underlines the fact that the O/Ti ratio and defect disorder play an important role in the electrical properties of TiO_2_ [50]. The dominant type of defect depends on the synthesis conditions, whether the films are prepared under reducing conditions and low temperatures or under oxidizing conditions and high annealing temperatures. It has been shown that the first technological conditions favor the formation of oxygen vacancies, while the second ones favor titanium interstitials [52]. It has been established that oxygen vacancy formation is more favorable in anatase crystal structures than in rutile ones [53]. Double ionized oxygen vacancies (V_O_^2+^) create localized donor states in the TiO_2_ bandgap, about 0.75–1.18 eV below the conduction band (E_C_), as detected by various measurement methods [47,54,55,56].

By capturing electrons, titanium vacancies (V_Ti_) are the only negatively charged ions, and thus, they are an acceptor-type of intrinsic defects. Although titanium vacancies are the minority, if their number is high enough, they can induce the switch from n-type to p-type TiO_2_. Wang et al. [57] demonstrated such a transformation in undoped anatase TiO_2_ films synthesized using a solvothermal method, where they were able to deposit p-type TiO_2_ films by introducing a large amount of V_Ti_ defects, up to 9.5 mol %. In this way, they obtained stable p-type non-stoichiometric TiO_2_ layers with significantly improved charge mobility and catalytic performance required for photoelectrochemical water splitting, pollutant removal, etc.

Another structural factor that has a strong influence on the electrophysical properties of TiO_2_ is the boundaries of crystalline grains. Interfaces form at the grain boundaries (GBs) that are in contact, creating electrostatic potential barriers (otherwise known as Schottky barriers), which in turn hinder the flow of the majority carriers and, due to their attractive potential, provide recombination centers for the minority carriers. The trapped charges at the grain boundaries influence the charge carrier transport properties. This effect is well known in polycrystalline semiconductors [58,59,60]. Yan Wang et al. [35] reviewed fourteen methods for the deposition of nanostructured TiO_2_. Although the materials obtained by these methods have different crystal phases, they all possess large surface areas and good electron transport properties, which allow more intense separation of photo-generated holes and electrons. To utilize the considered technologies, the processes behind the formation of TiO_2_ nanostructures by these methods are discussed in detail [35].

In most semiconductors, due to the presence of impurities or additives, the grain boundaries are electrically charged and strongly affect the electrical properties of the given structure. For nanostructured semiconductors, such as TiO_2_, the different charge transport mechanisms can be explained by grain boundaries, heterojunctions, Schottky barriers, or surfaces [61]. When the influence of deep energy levels and interface electric fields is taken into account, the electron transport mechanisms can be characterized via a simple Schottky double barrier model [61].

Depending on the type and degree of crystallinity, the relative dielectric constant (ε) of TiO_2_ can vary within a wide range of 23–170 [40,62,63]. The large dielectric constant and high resistivity make this material useful in the field of high-*k* dielectrics for electronics and could be successfully integrated in Si devices. For example, metal–oxide–semiconductor (MOS) structures formed with TiO_2_ oxides have good Si/TiO_2_ interface properties (interface state densities are of the order of 10^11^ cm^−2^eV^−1^, which is comparable to those of Si/SiO_2_), which confirms that TiO_2_ is a suitable alternative for CMOS applications as a dielectric [64]. Additionally, high-quality TiO_2_ films, deposited by the sol–gel spin method on Si substrates, had significantly low gate leakage currents in the formed Si MOS devices [65,66]. The study of the electrical characteristics of Si, InAs, and CNT field-effect transistors (FETs) with SiO_2_, Al_2_O_3_, HfO_2_, La_2_O_3_, and TiO_2_ as gate dielectrics and a detailed comparison of the short-channel parameters show that TiO_2_ has the best gate dielectric properties [67].

However, the high resistivity and low conductivity of TiO_2_ are a real drawback for other applications where photo-induced processes are essential, such as photovoltaic cells or photocatalysis. Extensive studies have been conducted to decrease the resistance and increase the conductivity, respectively, by improving the mobility of charge carriers in TiO_2_ to meet the requirements of each application. Since in the energy bandgap of pure TiO_2,_ almost all charges are compensated, the appearance of energy levels associated with impurities and defects in the structure may contribute significantly to the carrier conduction. Accordingly, to enhance the conductivity of the films, intensive research has been performed in two main directions: (i) the creation of a strong defect disorder introducing intrinsic defects in the TiO_2_ matrix, and (ii) introducing impurities by doping TiO_2_ with different metallic or non-metallic atoms. In both directions, intra-band states in the TiO_2_ bandgap are created, which play an important role in carrier recombination and transport mechanisms. Besides the crystal structure (polymorphic phases, size, and degree of crystallinity) and deposition methods and their conditions, intra-band states are a major factor determining the electrical properties of TiO_2_.

**Figure 6:**
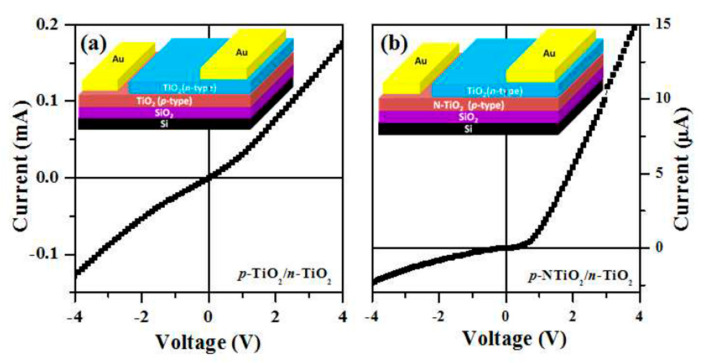
I–V characteristics of p-n homojunction of (**a**) p-TiO_2_/n-TiO_2_ and (**b**) p-NtiO_2_/n-TiO_2_ devices. The device architecture in both cases is shown as insets. Reprinted with permission from [41]. Copyright © 2016 American Chemical Society.

(i) In defect chemistry, the fabrication of pure and highly non-stoichiometric TiO_2_ films using various technologies was proposed to improve the transport properties of pure TiO_2_. Extensive studies have been conducted to tune the properties of TiO_2_ by creating oxygen deficiency in the TiO_2_ lattice [41,49,50,54,55,57,68]. Vasu et al. [41] synthesized pure anatase TiO_2_ films, p-type and n-type by nature, using the atomic layer deposition (ALD) method, but the resulting p-TiO_2_/n-TiO_2_ junction showed weak rectification behavior (Figure 6a). In order to improve the rectification effect, they were forced to dope the ALD p-TiO_2_ layer with nitrogen (Figure 6b). The N dopants generated V_Ti_ defect states (Figure 4b in Section 2.3), resulting in increased p-type conductivity and the appearance of strong room-temperature ferromagnetism in these films. This and many other experiments confirm that in order to synthesize stable p-type TiO_2_, acceptor-type impurities must be introduced into the films [38,41,56,69].

**Figure 7:**
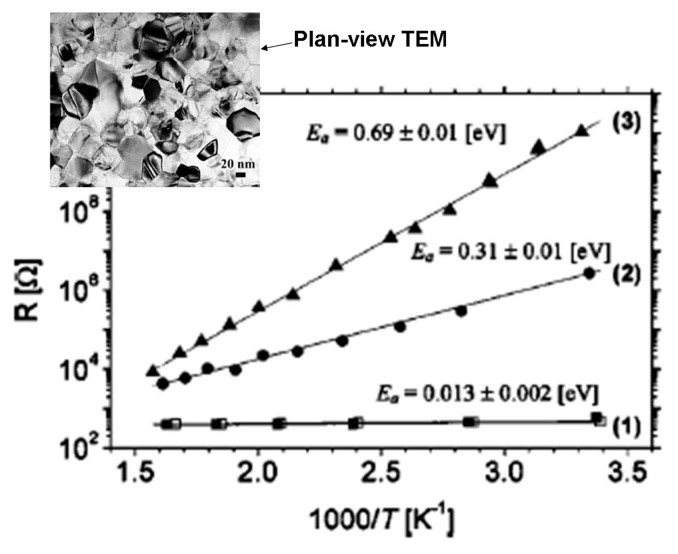
Resistance as a function of the reciprocal temperature at three different air pressures: (1) in vacuum (~4 × 10^−6^ mbar) (□—first set and ■—last set of measurements); (2) at 1 mbar of dry air (●); (3) at 10 mbar of dry air (▲). Ea is the activation energy. An inset plan-view TEM micrograph of a typical nc TiO_2_ film with an average of ~36 nm grain diameters is shown. Reprinted from [68] with the permission of AIP Publishing.

Rothschild et al. [68] presented a comparative study on rutile nanocrystalline TiO_2_ films annealed either in vacuum (reducing condition) or in dry air (oxidizing condition), monitoring in situ the behavior of the conductivity and I–V characteristics as a function of oxygen pressure and temperature. They found that the film annealed at 350 °C in dry air (at 10 mBar) had much higher resistance values and a larger surface potential barrier than that annealed in a vacuum (~4 × 10^−6^ mbar) (see Figure 7, where the resistance values were measured under equilibrium conditions). In the measured voltage range of (0.01–5 V), the current-voltage (I–V) characteristics of the reduced film were linear, while for the oxidized film, they were non-linear. Such non-linear behaviors of the I–V dependence are associated with charged grain boundaries (GBs) that control the charge transport mechanism. The authors in [68] suggest that potential barriers induced by oxygen chemisorption form at the surface and grain boundaries inside the film that control the charge transport in oxidized films. Vacuum annealing diminishes these barriers and makes the reduced film quite conductive (curve (1) in Figure 7). The observed effects are reversible and suggest that such nanocrystalline TiO_2_ films may serve as sensors for oxygen and gas sensing [68].

(ii) The introduction of impurities in the TiO_2_ matrix changes the structure and properties of the films and especially affects their electrical properties. In Section 2.3, the influence of doping impurities on the TiO_2_ bandgap value and, correspondingly, on the optical properties are discussed and highlighted with some reported experimental data.

The incorporation of dopant atoms into the TiO_2_ matrix generates defects that are responsible for the appearance of intra-band energy states (shallow or deep) in the TiO_2_ bandgap. These levels, which trap electrons or holes, act as recombination centers and strongly influence the charge transport properties of doped TiO_2_ films. If the dopant states give shallow energetic levels, the quasi-Fermi level moves closer to the E_c_ or E_v_ band edges, increasing the n-type or p-type conductivity, respectively. If they are deep levels, the quasi-Fermi level moves away from the E_c_ or E_v_ edges, respectively, reducing the current and Increasing the specific resistivity of TiO_2_. From all this, it follows that the current through the film proceeds by trapping and releasing charge carriers from the intra-band levels.

**Figure 8:**
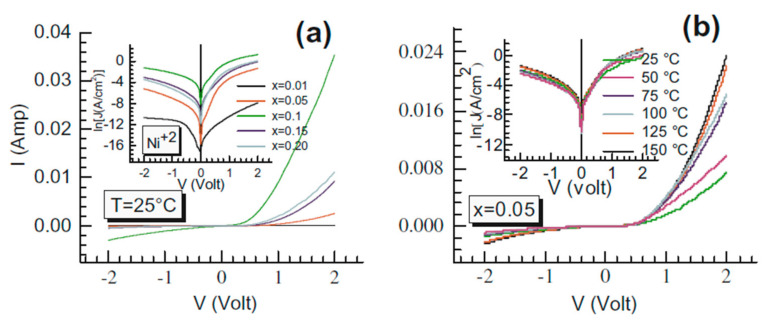
Current-voltage response of the n-ZnO/p-TiO_2_(Ni) heterostructures (**a**) at room temperature for different Ni doping concentrations and (**b**) with a concentration of x = 0.05 molar fraction at different temperatures. The inset of each figure shows the corresponding lnJ vs. V characteristics. Reprinted from [70] with permission from Elsevier.

In [70], it was demonstrated that by doping the TiO_2_ layers with Ni metal atoms, the forward current through the n-ZnO/TiO_2_(Ni) heterostructure was significantly increased in magnitude, while the reverse current was negligibly small (see Figure 8), proving p-type conductivity in the TiO_2_ film. The current-voltage (I–V) characteristics measured at room temperature of the ZnO/TiO_2_(Ni) heterostructures at different Ni doping concentrations are presented in Figure 8a, while Figure 8b shows the temperature dependence of the I–V characteristics of these heterostructures at a constant Ni molar fraction of x = 0.05. The corresponding I–V characteristics, given in a semi-logarithmic plot (inset in Figure 8a,b), well illustrate the strong increase in the current with an increasing Ni doping level and its much weaker dependence on temperature in the studied range of 25–150 °C. The performance of the device in [70] revealed that reproducible p-type conductivity can be obtained with Ni^+2^-doped TiO_2_ and can be effective in microelectronics and other energy-saving devices.

In [47], a comprehensive review of the possible impurities (nearly 40) of TiO_2_ doping for dye sensitized solar cell (DSSC) applications was performed. The contribution of each element to improving the device’s performance is summarized in Table 1 of the article. The authors emphasize the importance of finding the proper dopants that create shallow levels located close to the E_c_ band edge. This will avoid the transformation of these levels in recombination centers or their charge causing a large negative shift in the flatband voltage of the device, which would hinder charge injection.

To control the recombination processes and discover the role of shallow and deep levels in TiO_2_ films, a detailed characterization of their electrical properties, particularly the charge transport mechanism, is required. Therefore, the charge transport through the films prepared by different methods and doped with different types of impurities has been intensively investigated [38,40,45,52,62].

Due to the complex dependence of the structure and properties of TiO_2_ films on the preparation methods and the type of dopant impurities, the conduction mechanism in TiO_2_ films could be quite different and must be investigated separately for each specific device. Nevertheless, from the numerous research observations performed on this object [56,63,68,71,72,73,74], the general behavior of electron transport in TiO_2_ can be described.

Because of the charge compensation effect in TiO_2,_ the contribution of intrinsic free carriers to the conductance is negligible, even at higher temperatures (more than 300 K). Therefore, the observed charge carrier transport in this material mainly occurs through trapping and the release of charges from the energetic levels created in the TiO_2_ bandgap by intrinsic and/or extrinsic defects. At temperatures higher than 300 K, electronic transport is generally interpreted in terms of thermally activated conduction [71,73]. By lowering the temperature below 300 K, the current proceeds through the variable range hopping (VRH) carrier transport mechanism proposed by Mott [73] and further developed by Efros–Shklovskii (ES) [74]. The second model assumes that at the Fermi energy level (E_F_), a soft Coulomb gap forms and carrier transport is possible due to different hopping processes that are activated in different temperature ranges [74]. It has been experimentally confirmed that there can be a Mott-type VRH at higher temperatures and an ES-type VRH conduction at lower temperatures [72,75]. In the presence of deep levels in the TiO_2_ bandgap, the tunneling of charge carriers occurs from the occupied deep levels to the conduction or valence band of the films (trap-assisted tunneling) or to the nearest unoccupied deep levels (inter-trap tunneling) [56,68,71,75]. Inter-trap tunneling prevails when the inter-trap distance is smaller than the charge carrier path from the occupied deep level to the conduction band (E_c_) or valence band (E_v_). In Section 3, where the applications of TiO_2_-based structures in photocatalytic devices are discussed, the role of infra-band states (shallow or deep levels) is well illustrated. In this section, an overview of the factors that determine the electrical characteristics of TiO_2_ is outlined. This was supported by examples taken from the cited reports mentioning the application areas of each kind of studied TiO_2_-based structure. It was shown that the electrical properties of TiO_2_ material can be modified by introducing either defect disorders or dopants in the TiO_2_ matrix, according to the request of a given application area. In the next two sections, the TiO_2_-based structures, their application as photocatalysts and sensors, and the main results will be discussed in more details.

## 3. TiO_2_ as Photocatalyst

This section is mainly focused on highlighting some selected research related to those photocatalytic applications that help improve the quality of the modern life of society and serve for health and environmental protection. Here, we review the photocatalytic processes taking place in water disinfection, wastewater treatment, and self-sterilizing coatings. They are supported by examples taken from the available research.

Photocatalysis is an advanced oxidation process that removes traces of organic pollutants (at ppm or ppb level) from water or air under irradiation. It requires the use of advanced photocatalysts that are non-toxic, easily available, chemically and mechanically stable in aqueous environments, and are active under (preferably) solar irradiation. During photocatalysis, there are several steps that take place, which are as follows:

- The pollutant molecules are adsorbed on the photocatalytic surface. This is not dependent on irradiation. The photocatalyst is placed in contact with the pollutant solution (with or without stirring) in the dark until an adsorption–desorption equilibrium is reached. Adsorption can be reinforced by pH control of the medium in order to favor electrostatic interactions between the pollutant molecules and the photocatalyst.
- Upon activation of the photocatalyst by irradiation, electrons from the valence band jump to the conduction band, leaving behind holes. This step proceeds only if the radiation source energy is higher than the bandgap energy of the photocatalyst. Usually, semiconductor oxides have good photocatalytic properties. TiO_2_ is the most commonly used for this purpose.
- The formation of reactive oxygen species (ROS) occurs when the holes from the valence band react with previously adsorbed water molecules on the surface of the photocatalyst and form highly reactive hydroxyl radicals. At the conduction band level, the electrons react with the previously adsorbed oxygen molecules, leading to the formation of highly reactive superoxide species.
- The created ROS then attack the pollutant molecules, causing them to break down into smaller organic compounds, ideally CO_2_ and H_2_O (mineralization).
- Finally, the decomposition products are desorbed from the photocatalyst surface, and the process can start over again.

Briefly, the TiO_2_ photocatalysis process can be summarized as the generation of electrons and holes upon the absorption of photons, the capture of photogenerated electrons, and the redox reaction on the surface of TiO_2_, leading to the decomposition of the pollutants [1].

TiO_2_ has been widely accepted as the most active photocatalyst that meets the criteria of high stability under different reaction conditions, resistance to photo-corrosion, and high photo-activity. It has been extensively studied because of its advantageous properties, such as high chemical stability in aqueous environments (over a large pH domain), non-toxicity, and low cost. One of the main disadvantages of titanium dioxide is the fact that, due to its wide bandgap energy of 3.2 eV, it can only be activated under UV irradiation, which accounts for only a small part of the solar spectrum. Another disadvantage is the fast recombination of electrons and holes, which can be delayed by coupling TiO_2_ with other materials, such as metal oxides or carbon derivatives, in order to extend the charge carrier lifetime, allowing them to form ROS. In order to extend the activation domain, TiO_2_ needs to be doped with metal or non-metal ions or to be coupled with other semiconductors with appropriate band alignment.

Recently, nanohybrids obtained by incorporating graphene into a TiO_2_ nanostructure have been proposed to reduce the bandgap energy while minimizing the inherent drawback of electron pair recombination in TiO_2_. In [1], such a solution to the mentioned problems has been implemented. The incorporation of TiO_2_ nanoparticles in 2D nanomaterials considerably reduced the bandgap energy of the prepared TiO_2_ nanohybrids compared to the pristine TiO_2_ and decreased the recombination rate of photo-generated electrons and holes during the photocatalytic activity. In addition, it provided a large surface area that promoted higher photocatalytic activity.

There is also a discussion on whether to use powder or film in the photocatalytic process. Powders have a much higher surface area, allowing better adsorption and degradation of the pollutant. However, they are much more difficult to recover from water and more difficult to reuse. Thin films compensate for their smaller surface area by being very easy to reuse. Another solution to this issue could be to deposit thin films on different, smaller surfaces (such as beads), as this will result in a large surface area but also easier recovery. Another solution is to use TiO_2_ nanoparticles or nanostructured TiO_2_.

Acting as a photocatalyst, TiO_2_ can be used in applications such as water disinfection, wastewater treatment, self-sterilizing coatings, self-cleaning coatings for the built environment, food preservation, etc. There are many other areas of application, but in this review, we have chosen to present the most significant updates of the past 5 years in the fields of water disinfection, wastewater treatment, self-sterilizing coatings, and gas and biosensors.

### 3.1. Water Disinfection

Access to clean drinking water is difficult in many countries, and the risk of waterborne disease transmission can be very high. One of the most common bacteria that humans are faced with is *Escherichia Coli* (*E. coli*). *E. coli* is used as an indicator of fecal pollution in water; it is relatively stable and difficult to remove, and therefore, it is often used as a model bacteria in the research of novel materials and technologies for water disinfection.

Titanium dioxide and TiO_2_-based composites have been shown to exhibit antibacterial activity under certain conditions, both against *E. coli* and other microorganisms.

Monteagudo et al. [76] reported on the use of TiO_2_ P-25 Evonik powder immobilized on the borosilicate tubes of an annular continuous-flow compound parabolic collector reactor (1 L volume) with a pasteurization system (0.5 L) incorporated. With a load of 0.60 mg/cm^2^ of TiO_2_, 99.1% bacteria photoinactivation was reached after 80 min under solar irradiation of 10–27 W/m^2^ in synthetic water that also contained antipyrine. The effect of HO• radicals on the overall disinfection rate was outlined as the addition of 150 mg/L H_2_O_2_ leading to the generation of 2000–3000 nmol/L hydroxyl radicals [76].

In [77], the inactivation of *E. coli* using commercial anatase TiO_2_ powder provided by Shanghai Macklin Biochem Co., Ltd. (Shanghai, China) (1 g/L) under UV-LED lighting (4.9 W/m^2^) is reported. It was shown that the disinfection rate was dependent on the radiation wavelength, as at 265 nm, the highest inactivation efficiency was reached. Ilaria De Pasquale et al. in [78] reviews the use of TiO_2_ in the treatment of the SARS-CoV-2 virus. Khaiboullina et al. [79] have also reported the successful use of TiO_2_ nanoparticles and nanotubes in the deactivation of the HcoV-NL63 and the SARS-CoV-2 viruses, respectively.

The antibacterial activity of TiO_2_ coatings can be intensified by increasing the temperature of thermal treatments and the formation of the anatase crystalline structure. However, if the thermal treatment is too long, the rutile crystalline structure is formed, and the antibacterial activity decreases. The doping of TiO_2_ coatings with Ag can maintain the anatase structure and prevent the formation of the rutile phase.

By doping the TiO_2_ structure, activation in the visible spectral range can be achieved, and therefore, the overall cost of the disinfection process can be reduced. It has been known since ancient times that water disinfection can be performed by placing a Ag object in the water.

A review from 2021 by He et al. [9] looked at the reports on water disinfection using TiO_2_ loaded with noble metals such as Pt, Au, or Ag, as well as composites of TiO_2_ and carbon nanomaterials. It was shown that thin films, nanotubes, and nanofibers of noble metal-loaded TiO_2_ were used for the inactivation of *E. coli* from water under different types of radiation (solar, UV, fluorescent, etc.), with process durations of anything between 1 h and 24 h [9]. One of the main concerns when using these materials is the leaching of the metal ions into the water, though few reports focus on this.

Wu et al. reported on hydrothermal Ag-doped TiO_2_ nanofibers [80]. The doping level and the calcination process were varied to optimize the photocatalytic properties of the composites. At 5 mol% Ag and after 600 °C 12 h thermal treatment, the nanofibers acquired high inactivation rates for both *E. coli* and *S. aureus* when exposed to VIS radiation. The authors suggest that the disinfection is mainly due to the superoxide radicals that damage the DNA/RNA of the bacteria rather than the hydroxyl radicals, which are not produced due to the unsuitable band alignment toward redox potential [80].

Gadgil and Vidya Shetty [81] have reported on Ag-TiO_2_/PANI composites containing 13% Ag that, under VIS irradiation for 2 h, could completely disinfect water containing 50 × 10^8^ CFU/mL *E. coli*. The optimized photocatalyst load was 1 g/L. Both photocatalytic water disinfection and endotoxin degradation were reported. The Ag-TiO_2_/PANI composites could be used both in free and immobilized forms.

Another emerging trend is the incorporation of green chemistry in the synthesis of novel composite materials. Recently, Torres-Liminana et al. [82] reported on a Ag-TiO_2_ composite using Eucalyptus globulus as the Ag source. In parallel, they also reported conventionally prepared Ag-TiO_2_ composites using the more toxic reagents NaBH_4_ and AgNO_3_ for sol–gel (SG) and microwave-assisted sol–gel (MWSG) processes. The composite material of anatase TiO_2_ and Ag nanoparticles, obtained by MWSG, was able to completely inactivate both *E. coli* and *S. aureus* in the absence of any light source. The results of green synthesized composites and those of composites obtained by the traditional deposition methods are comparable [82].

The Cu-doped sol–gel/TiO_2_ material [83] is also a good inhibitor of *E. coli* under visible light due to the strong oxidizing reactive oxygen species destroying the bacteria.

Alongside metal doping, there are also reports on TiO_2_ doped with non-metal atoms. One of the most frequently used dopants is nitrogen. Pablos et al. [84] obtained N-doped TiO_2_ nanotubes (NT) by anodizing Ti foil, as well as growing nanoparticles (NP) in N-doped TiO_2_ on Ti foil by the sol–gel process. The NT films were more efficient in the electrochemically assisted photocatalytic inactivation of *E. coli* under UV/Vis irradiation compared to the NP ones. This was attributed to the NT structure, offering a less resistant path for the electrons to travel, thus leading to reduced electron–hole recombination. Both sets of samples showed negligible activation when irradiated only with VIS light [84]. Makropoulou et al. in [85] discussed the influence of the nitrogen source (urea, triethylaminem and NH_3_) on the composite activity against three types of bacteria, *Escherichia coli*, *Pseudomonas aeruginosa*, and *Bacillus cereus*, under artificial sunlight. It was shown that the bactericide effect of the composites followed the order N-TiO_2_ (Urea) > N-TiO_2_ (NH_3_) ~ N-TiO_2_ (TEA) > synthesized TiO_2_ > TiO_2_ P25. Moreover, the *E. coli* and *P. aeruginosa* bacteria were completely inactivated during 1 h of treatment when starting at 10^6^ CFU/mL and using 50 mg/L of the photocatalyst [85]. The *B. cereus* was much harder to remove, and complete elimination could be reached within 1 h only when using N-TiO_2_ (TEA) at 100 mg/L. They also confirmed the findings of [84] that VIS irradiation does not activate the composites.

By combining two (or more) metal oxides with an appropriate energy band alignment, composites with lower bandgap energy and hence VIS activation can be obtained. Some examples include heterojunctions based on TiO_2_ and SiO_2_, FeO_x_, CeO_2_, etc.

The research group of Levchuk et al. [86] obtained TiO_2_/SiO_2_ thin films on flexible PET substrates using the material printing technique. They used these in a continuous flux photoreactor under natural sunlight to disinfect (1) drinking water contaminated with *E. coli* or *Enterococci*, as well as (2) seawater contaminated with *Vibrio owensii*, *Vibrio alfacsensis*, and *Vibrio harveyi.* They found that bacteria adherence to the hydrophobic thin films was higher than to the hydrophilic ones, leading to higher disinfection rates for the former. Inactivation of *E. coli* was performed at a higher rate than *Enterococci* in the drinking water. Saltwater led to the inactivation of the photocatalyst (after 10 cycles) due to the deposition of salt ions from the seawater on the TiO_2_/SiO_2_ surface. Ultimately, the authors acknowledged that there is room for improvement in the thin films.

In [46], the deposition of TiO_2_-CeO_2_ nanotube arrays on Ti mesh through anodization followed by electrodeposition was described. These structures were reported to be effective in the disinfection of water infected with *E. coli* and *S. aureus*, with CeO_2_ increasing the spectral response range to a certain extent and promoting the separation of the electrons and holes. The amount of CeO_2_ nanoparticles coated on the TiO_2_ nanotubes must be controlled, as an excessive amount can prevent light absorption by the latter, leading to a reduction in the inactivation rate of the *E. coli* bacteria [46].

Other composites based on TiO_2_ have also been proposed as a solution to easily retrieve the photocatalyst powder from the solution to reuse and recycle. Therefore, Keeley et al. [87] reported on a sol–gel Fe_2_O_3_-SiO_2_-TiO_2_ composite that led to the complete inactivation of *E. coli* (initial concentration 10^4^ CFU/mL) from distilled water under UV light and within 25 min. The photocatalyst was also successful in the disinfection of a real surface water sample containing 500–5000 CFU/mL of different microbes with a 62 ± 3% bacterial count reduction within 30 min. The authors also reported on the influence of pH on the amount of TiO_2_ nanoparticles that can be loaded onto the gel Fe_2_O_3_-SiO_2_ composite. A more alkaline environment (pH = 10) was reported to lead to a higher TiO_2_ loading compared to neutral or even acidic environments (pH = 3).

Graphene–oxide TiO_2_ composites have antibacterial properties under solar irradiation, and they can improve the photocatalytic disinfection performance in wastewater, acting in the following ways:

- To generate the long-lifespan reactive species (•O_2_^−^ and H_2_O_2_);
- To enhance the interaction between the bacteria and the photocatalyst;
- As photocatalysts in particle form to provide more reactive surfaces for contacting bacteria.

**Figure 9:**
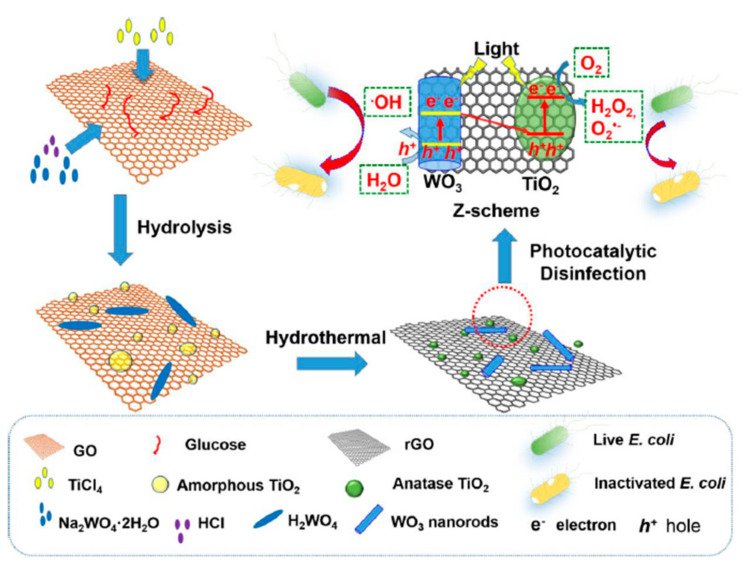
Illustration of the synthesis of TRW and its application as Z-scheme photocatalysis system for water disinfection. Reprinted from [88] with permission from Elsevier.

A triple-composite oxide based on TiO_2_ nanocrystals, WO_3_ nanorods, and rGO forming a Z-scheme heterojunction was reported in [88]. The synthesis is schematically represented in Figure 9. This composite powder (in a concentration of 1 mg/mL) was tested under simulated solar radiation (using a 200 W arc Mercury-Xenon lamp with an AM 1.5 filter) and showed improved bacterial (*E. coli*, 2 × 10^3^ CFU/mL) inactivation compared to the TiO_2_-WO_3_ composite after 80 min. The authors attributed this to rGO acting as an electron scavenger and suppressing the recombination of electron–hole pairs, as well as boosting the O_2_ reduction reactions during the photocatalytic process.

Berberidou et al. [89] reported on TiO_2_-rGO composites with 1–10% rGO that were used to inactivate *Bacillus Stearothermophilus* (at an initial concentration of 10^5^ or 10^6^ CFU/mL) under UV-A illumination. The highest inactivation rate was recorded for the composite containing 5% rGO, which led to a full disinfection within 2 h. The detection of genomic DNA in the suspension suggested an oxidative attack of the reactive oxidative species on the outer and inner coats, followed by lysis and spore death.

**Figure 10:**
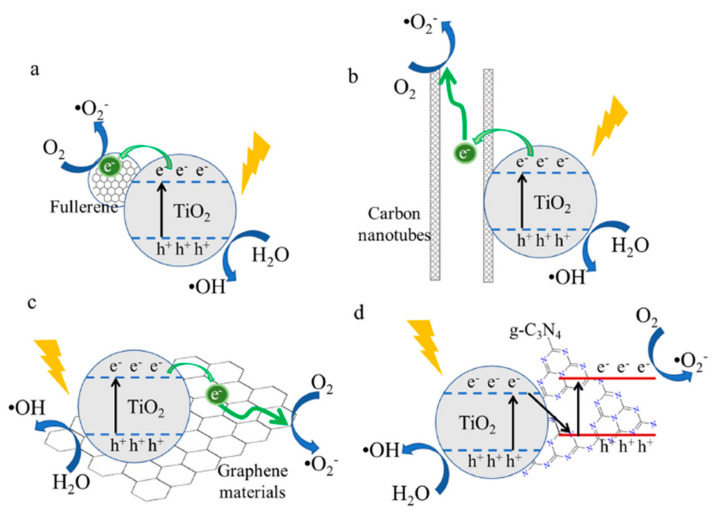
Schematic illustration of the photocatalysus mechanism of carbon nanomaterial–TiO_2_ composites: (**a**) fullerene/TiO_2_ composite; (**b**) carbon nanotube/TiO_2_ composite; (**c**) graphene material/TiO_2_ composite; and (**d**) g-C_3_N_4_/TiO_2_ composite. Reprinted from [9] with permission from Elsevier.

In a previously mentioned paper [9], He et al. reported on composites based on TiO_2_ and carbon derivatives, which have been gaining much more attention in recent years. The authors proposed different photocatalytic mechanisms for different carbon derivatives, such as fullerenes, carbon nanotubes, graphene materials (graphene, graphene oxide, reduced graphene oxide, etc.), and g-C_3_N_4_ (Figure 10). It is important to note that, in all cases, the carbon derivative acted as an electron scavenger from the conduction band of the titania, thus prolonging the lifetime of the charge carriers by delaying the recombination of electrons and holes. This also means that the formation of hydroxyl radicals took place at the TiO_2_ level, whereas the formation of superoxide took place on the carbon derivative. There are, of course, some drawbacks to using carbon derivatives, such as the following:

- Fullerenes are hydrophobic, and therefore, the formation of TiO_2_–fullerene junctions is not easy;
- CNT may aggregate during the composite synthesis, limiting the available surface for TiO_2_ grafting;
- Graphene is only stable around 160 °C, and therefore, it can lead to the formation of low-degree crystalline composites;
- g-C_3_N_4_ is, so far, the most promising carbon derivative, as it maintains its 2D structure even at high temperatures and does not release any toxic elements into the environment.

### 3.2. Wastewater Treatment

The use of titania in wastewater treatment is well known and reported, as TiO_2_ is still considered the benchmark photocatalyst due to its high activity, high chemical stability in aqueous environments, low cost, and overall low toxicity. Due to this fact, titanium dioxide and materials based on it are often investigated for the removal of organic pollutants from wastewater. The source and toxicity of these pollutants vary significantly, but an interesting category is that of materials (or traces of materials) coming from the medical field. From wastewater, these can move to natural streams, infiltrate into the ground, and then be further taken up by plants and vegetables. Even if traces of such organics are allowed into wastewater streams, there is a high risk of bioaccumulation in flora and fauna, even humans. If they are not toxic, these substances can still pose a risk to the health of humans, as, over time, the body becomes immune to the active ingredients in medicine.

Another issue in benchmarking is setting the protocol by which materials can be tested and compared against one another. This is a difficult aspect in most fields, and research is being performed to solve the particular issues, but seldom are the testing conditions (photocatalysis duration, light source choice (radiation intensity and spectral range) and position, photocatalyst load, photocatalyst to pollutant ratio, etc.) similar. Therefore, it has been agreed to use a standard that involves methylene blue as the pollutant at an initial concentration of 10 μmol/L under 10 W/m^2^ UV exposure. This standard (ISO 10678) is sometimes modified, but it should be referenced in order to facilitate a comparison between the performance parameters of novel materials. Therefore, this review will focus mostly on novel materials used in the wastewater treatment of methylene blue.

Dey et al. [90] obtained anatase TiO_2_ through continuous and pulsed plasma-enhanced chemical vapor deposition at substrate temperatures below 60 °C. The thin films were deposited on thick polycarbonate plates and tested in the photocatalytic degradation of methylene blue in an aqueous solution (initial concentration of 1 μmol/L) under 20 W/m^2^ UV exposure as well as under simulated solar radiation. Under simulated solar radiation, the anatase-coated substrate showed good ability for Mb degradation, leading to an apparent rate constant degradation of 0.4 h^−1^ [90].

Recently, Covei et al. [91] deposited sol–gel TiO_2_ thin films on glass beads to remove the methylene blue from water by photodegradation in a mix light source (UV/Vis). The substrate form and its preparation before TiO_2_ deposition, sol dilution, and the thickness and roughness of TiO_2_ layers were carefully monitored by checking the structure, the morphology, the chemical composition, and the photocatalytic performances of the prepared layers [91].

By doping TiO_2_ with 5% Ag, Sing et al. [92] showed that a solar-activated photocatalyst for the degradation of methylene blue can be obtained.

In the noble metal category, Pt and Au can also be added to TiO_2_ for water disinfection. Scarisoreanu et al. [93] prepared nanospheres of titanium dioxide decorated with Pt, Au, and Ag synthesized by laser pyrolysis combined with wet impregnation for the total photodegradation of methyl orange in aqueous solutions under UV and Vis irradiation. The most rapid complete degradation was observed for the samples containing Ag. Their spherical morphology contributed to maximum active surface contact. Anatase/rutile coupling and noble metals act on bandgap energy, but what is most important is that they are biocompatible with human lung cells.

The research group of Cao et al. [94] reported on Mg-doped TiO_2_ synthesized using template-free and surfactant-free solvothermal methods. The Mg-to-Ti molar ratio was varied between 0:1 and 0.014:1. The tests were performed on rhodamine B, starting at the initial concentration of 10^−4^ mol/L with a catalyst load of 10 g/L. The most promising photocatalytic films (with Mg:Ti = 0.01:1) showed a high photodegradation rate of methylene blue of 99.5% in 80 min exposed to visible light irradiation, which proved significantly higher than the degradation rate of pristine TiO_2_ [94].

Mahanta et al. [95] reported an efficient SiO_2_-TiO_2_ nanoparticle system that has good photocatalytic efficiency in methylene blue degradation due to the presence of highly crystalline anatase TiO_2_ as well as a good adsorption capacity due to SiO_2_-induced porosity.

Another oxide that is commonly coupled with TiO_2_ is ZnO. It was reported that TiO_2_-ZnO nanocomposites showed photocatalytic activity under visible irradiation and could lead to up to 96% degradation of methylene blue after 3 h, which was significantly higher than using TiO_2_ or ZnO individually [96]. Similar good results were also reported by [97] regarding α-Fe_2_O_3_/TiO_2_ sol–gel nanocomposites, which were used to degrade methylene blue (photocatalytic efficiency of 90%) and phenol (photocatalytic efficiency of 50%) under solar-like irradiation (500 W) after 3 h.

Graphene is one of the most widely used 2D nanomaterials with high electrical mobility (2 × 10^5^ cm^2^V^−1^s^−1^), high surface area (2600 m^2^g^−1^), and high transmittance (97%) [98,99,100,101]. Due to these properties, graphene has been extensively used to obtain composite materials with TiO_2_ [1]. It acts as a charge scavenger for the electrons in the TiO_2_ conductance band, delaying the recombination between photo-generated electron and hole pairs. It can also increase pollutant adsorption due to its high surface area. Much like doping, the use of 2D materials can lead to a improved overall photocatalytic efficiency and solar activity [102,103,104,105,106,107].

TiO_2_-GO composites containing TiO_2_ nanoparticles obtained using green alga Chlorella pyrenoidosa and deposited on GO nanosheets were shown to exhibit higher photocatalytic efficiency compared to pristine TiO_2_ nanoparticles with three cycles of reusability. Crystal violet dye (40 ppm) and visible light were used [108].

An article by Covei et al. [109] reports on composite thin films based on TiO_2_, WO_3_, and rGO (50:50:1 wt) that proved photochemically active under UV/Vis irradiation of low irradiance (34 W/m^2^) against both methylene blue and phenol. It was shown that the higher photocatalytic efficiency obtained when using methylene blue (28% compared to 16%) is due to photocatalyst sensitization, which does not occur in the case of phenol. Elsewhere [110], the authors also reported on the stability of these thin films under different conditions, and it was found that high relative humidity (90%) affects their morphologic, optical, and photocatalytic properties more significantly than high irradiance (1000 W/m^2^) or high temperatures (40 °C).

### 3.3. Self-Sterilizing Coatings

A review from 2021 touched upon the methods by which metal nanoparticles can help inhibit viruses [111]. These include modifying the surface of the viruses in order to prevent attachment, the use of metal ions and ROS that can damage the viral genetic material, and breaking the dislfide bonds between amino acids in order to disable the viruses.

The review mentions multiple reports of TiO_2_ used as an antiviral coating, possibly even against the SARS-CoV-2 virus, which spreads in aerosolized droplets that settle on surfaces. It is proposed that the TiO_2_ nanoparticle-based surfaces could use the moisture from these droplets to start the redox chemical reactions to obtain reactive oxygen species that will then affect the proteins on the viral surface [111]. It is also proposed that SARS-CoV-2 inhibition could be achieved through oxidative damage to its base proteins, lipids, and nucleic acids, all due to •OH and O_2_^−•^ radicals [111].

Photocatalytic coatings have been synthesized using low-temperature oxygen plasma treatment of the polymer PP followed by the deposition of TiO_2_ nanoparticles and their sensitization with different organic ligands such as catechol, 2,3-naphthalenediol, pyrogallol, and rutin (concentration of 10 mmol dm^−3^) [112]. The films were deposited on ITO foil and photocurrent generation was observed, most significantly for the titanium dioxide coating modified with catechol. Two adsorption bands were found for the modified films: one in the UV range, which could be attributed to the adsorption of TiO_2,_ and a weaker one, which is attributed to the charge transfer band of the Ti(IV)-L surface complex. This last one is responsible for visible-light-induced photocurrent generations as well as photocatalytic reactions. Due to this finding, the authors recommend the use of these novel photocatalytic coatings in self-sterilizing applications [112].

**Figure 11:**
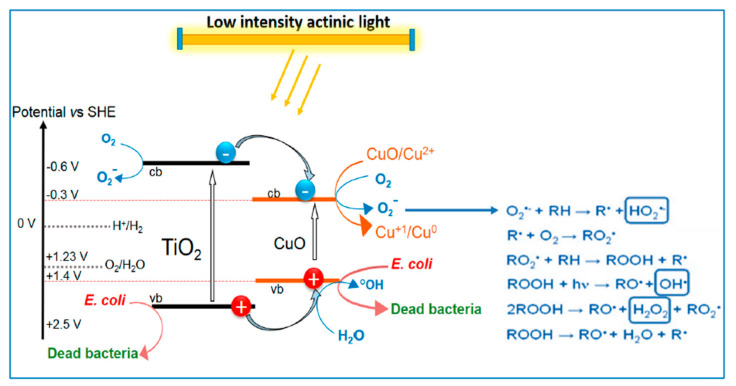
Diagram suggested for of bacterial inactivation under solar simulated light photocatalyzed by TiO_2_/Cu films sputtered on polyester (PES). Reprinted from [113]. Copyright © 2017 by Rtimi, S., Giannakis, S., and Pulgarin, C.

In a review by Rtimi et al. [113], it was suggested that the use of catalytic/photocatalytic textiles (for bedding, curtains, lab coats, and other objects used in hospitals) could decrease bacterial propagation. They highlight the advantages of sputtering methods for these cases over traditional chemical methods (such as sol–gel) for uniform, reproducible deposition resulting in mechanical stability and adhesion of the films to the substrate. In [113], TiO_2_ coatings with Ag and Cu dopants were deposited on glass and polymer substrates. They have antibacterial properties that are attributed to the formation of CuO, which promotes charge separation in TiO_2_, allowing the formation of superficial •OH radicals. Further CuO particles can be reduced to Cu_2_O by the charges generated in TiO_2_ under UV/Vis light exposure, and then they can be oxidized back to CuO [113,114]. This process is illustrated in Figure 11. In addition, binary complexes based on FeOx and TiO_2_ were also considered as promising materials for environmental decontamination and self-cleaning and self-sterilizing surfaces [113,114,115].

### 3.4. Self-Cleaning Coatings for Built Environment

Many authors presented new and innovative synthesis routes for producing photocatalysts with applications in self-cleaning and decontaminating coatings on stones and other building materials [116,117,118,119,120,121].

### 3.5. Food Preservation

The material containing TiO_2_ nanoparticles increased the antioxidant and antimicrobial ability from 51% to 71%, and it is considered a very stable material for food packaging [122,123,124,125,126,127,128,129].

## 4. TiO_2_ as Sensor

This chapter will discuss some aspects of gas sensors that warn us of the appearance of toxic gases and protect us from serious or even fatal illnesses, as well as biosensors that signal the early onset of chronic diseases and help restore the physiological state for the normal functioning of the living organism.

### 4.1. Gas Sensors

The detection and monitoring of toxic gases in the environment (in domestic as well as industrial areas) are highly significant to both personal and environmental safety [130].

**Figure 12:**
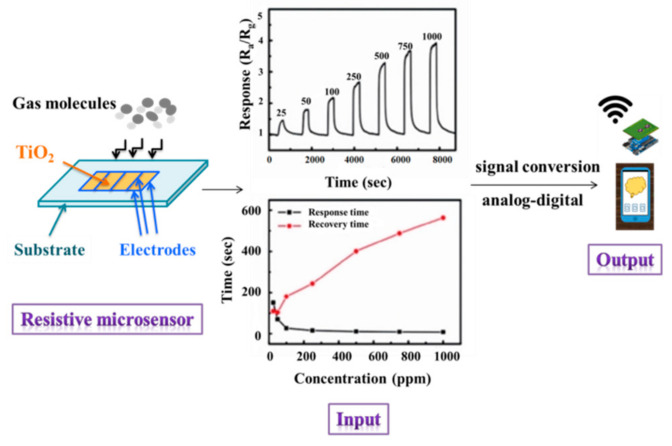
The principle of gas detection using a resistive sensor based on a transducer. Reprinted with permission from [131] (copyright © 2020 Paul Chesler and Cristina M. Vladut) and adapted from [132] (copyright © 2017 by André Decroly, Arnaud Krumpmann, Marc Debliquy, and Driss Lahem).

A gas sensor can be defined as a device consisting of a transducer and a sensitive film or membrane that generates a signal related to the concentration of a certain gas species. In Figure 12, the principle of gas detection using a resistive sensor is presented [131].

TiO_2_ is the third-most-used oxide in the field of sensors after SnO_2_ and ZnO. However, it has two major disadvantages: its high detection temperature, which is usually above 800 °C, and its large electrical resistance. Both can be overcome by introducing certain dopants into the TiO_2_ lattice.

The reaction mechanism of the sensors based on TiO_2_ involves two stages. The first stage takes place at a low temperature (below 300 °C), at which the gas physically adsorbs on the TiO_2_ surface. The second stage takes place at a higher temperature (above 300 °C), where the gas molecule catalytically oxidizes, consuming oxygen from the TiO_2_ lattice. During this second step, TiO_2_ ions are created when the gas molecules are oxidized and electrons are released. These are responsible for the decrease in electrical resistance, which constitutes the sensor response [133]. The regeneration of the sample can then be performed using the oxygen from the bulk of the TiO_2_.

In the following section, we try to summarize the gas sensors developed in the last 5 years based on TiO_2_ deposited on various substrates by chemical and physical methods.

As it can be seen from Table 2, the sensitive materials can be nanoparticles (doped and undoped), films, or composite materials of TiO_2_ with metals, graphene, and polymers. They can be deposited on glass, quartz, silicon, and alumina by physical (R.F. sputtering, DC magnetron sputtering, atomic layer deposition, pulsed laser deposition, etc.), chemical (sol–gel, hydrothermal, spin coating, pyrolysis, printing, chemical vapor deposition, and flame spray synthesis), or electrochemical methods, as well as by combined methods such as sol–gel and hydrothermal techniques (or microwave-assisted hydrothermal method).

The properties of a sensing film (sensitivity, stability, reproducibility, recovery, and low work temperature) essentially depend on the following:

- The substrate material, especially for the electrolytic deposition of TiO_2_. The main problem is the side reaction of water oxidation occurring at the substrate–electrolyte interface, releasing oxygen [132].
- The number and thickness of layers in the film.
- The preparation method would provide the porous structure and the rough surface of the films. It is well known, for example, that the SG is a chemical method that produces the most porous films.
- The type and concentration of the dopants/composites added to the TiO_2_ films.
- The preservation of the anatase phase after the consolidation of the film.
- The grain size must be small enough to significantly increase the surface area.

**Figure 13:**
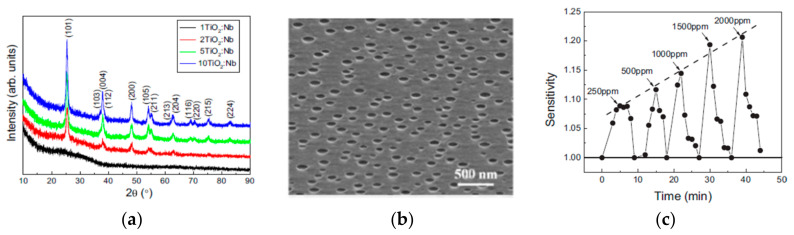
The main characteristics of Nb-doped 10-layer TiO_2_ film deposited on Si: (**a**) XRD spectra; (**b**) SEM cross-section image of the 10TiO_2_:Nb thin film; and (**c**) recovery characteristics of the film at an operating temperature of 400 °C. Reprinted from [149] with permission from Elsevier.

An example of all these is in Duta et al. [149], who argue the role of Nb doping as a donor in the TiO_2_ matrix to increase the number of available charge carriers. The analysis of the XRD spectra showed that the SG films deposited on glass substrates were anatase; no peaks related to the rutile phase were detected (Figure 13a). Figure 13b shows the SEM image of the 10-layered films, revealing the presence of shallow cavities, which lead to a higher surface area, which is an advantage in gas sensing [149]. These 10-layered Nb-doped TiO_2_ films exhibit good sensitivity for the wide-range detection of CO (0–2000 ppm) at the working temperature of 400 °C, as shown in Figure 13c.

For advanced chemical gas sensors, high sensitivity, stability, reproducible preparation, and selectivity are required. Selectivity is a major issue that can still be overcome by hybrid-sensitive materials. Some examples are provided below for the detection of NH_3_, which is one of the most harmful gases that affect the immune system when exposed to large amounts. Direct ammonia exposure can cause nose and throat irritation, coughing, and respiratory tract irritation.

Combinations of TiO_2_ with polymers, carbon derivatives, or inorganic nano-heterostructures are a good solution to overcome this serious issue. Such successful combinations are as follows:

- Tannin sulfonic acid-doped polyaniline–TiO_2_ composite [150];
- Cellulose/TiO_2_/PANI composite nanofibers that incorporate p-n heterojunctions [151];
- TiO_2_-NiO nanostructured bilayer thin films [152];
- Protonated porphyrin/TiO_2_ composite thin films [153];
- rGO-decorated TiO_2_ microspheres [154];
- TiO_2_/ZnO and ZnO/TiO_2_ core/shell nanofibers [155];
- PANI-TiO_2_-Au ternary self-assembly nanocomposite thin film [156];
- Graphene/titanium dioxide hybrid [157];
- PANI/TiO_2_ core–shell nanofibers [145];
- TiO_2_/SnO_2_ and TiO_2_/CuO thin film nano-heterostructures [141].

### 4.2. Biosensors

In the last decade, the rapid development of biosensor technologies has made them promising diagnostic tools in various fields of biology, microbiology, ecology, toxicology, medicine, etc. The advantages and disadvantages of TiO_2_ biosensors have been well summarized in [158]. Here, we review the types of biosensors that are closely related to medical applications. Despite the great progress of biosensors and the large number of publications in this field, we are still far from eradicating serious diseases.

A biosensor uses biomolecular interactions between a biorecognition element and the investigated substance (enzymes, complementary DNA, antigen, etc.). This produces a signal that is retrieved by a transducer [159].

The most important field of TiO_2_ biosensors is medicine (diagnosis, treatment, testing, biomarkers, etc.). Biosensors can be categorized in many ways, one of them being by detection type, such as the following:

- Fluorescent biosensors [160]. They rely on the photon emission of fluorescent substances when excited molecules (or atoms) give off energy during the relaxation stage [161].

In [161], the presence of defect levels generated by the oxygen vacancies is confirmed by the measured fluorescent spectra of TiO_2_ NPs, where emission bands in the UV/visible spectrum are observed at 274 nm—related to the deep donor level (oxygen vacancy) at 387 nm—being emissions due to the annihilation of excitons, and at 421 nm, 491 nm, 532 nm, 574 nm, and 612 nm—related to surface states.

Titanium dioxide is especially used in photodynamic diagnosis (PDD), where cancerous tissues can be identified through imaging [162,163,164]. TiO_2_ can scatter incident light, and therefore, a stronger signal can be obtained in fluorescence PDD [165].

- Field effect transistor (FET) biosensor [158]. The FET acts as a transducer for the signal obtained through the biointeraction of the biorecognition element and the biomarker, especially those from early-stage cancers [166]. Azizah et al. [167] reported on a biosensor for the detection of Human Papillomavirus (HPV), a precursor to cervical cancer [167].
- Plasmon resonance biosensor (SPR). Xu et al. [168] fabricated an SPR composed of titania modified with Au nano-island for the detection of glioma (a common type of tumor originating in the brain) cells [158].
- Aptasensors [169]. These sensors use artificial DNA or RNA sequences called aptamers to identify and bind specific molecules [170].
- Electrical biosensors [159] are based on the trapping of an analyte by the interface of an electrode, which generates an electrical signal (current, potential, capacitance, impedance, and resistivity). There are two major classes of electrical biosensors, namely amperometric [171,172] and impedance [173] biosensors. The former measures the electrical current emmited in redox reactions, whereas the latter measures the conductance or capacitance. They are used in microbiology to detect, correctly identify, and even quantify different types of bacteria.
- (Photo)electrochemical biosensors (PEC) [174,175,176,177] use photoelectrical materials as electrodes. These are able to convert light into electrical current that, when processed, is used for the detection and quantification of biomolecules. PEC sensors are sensitive and have good analytical performance, as well as a low background signal [178,179,180].

Wang et al. [181] prepared a PEC sensor for visible light composed of a heterojunction of titania and CdTe to replace NIR light and amplify the electrical current.

The biosensors can detect the following: cancer [139,182,183,184]; cholesterol [185]; COVID-19 [186,187], DNA [177,188,189,190]; dopamine [191]; and glucose with PEC [175,176,192,193], amperometry [194], mpedimetry [195,196], electrochemical [197], glucose oxidase film [198,199,200]; hydrogen peroxide (H_2_O_2_) [201,202]; lactose [203,204]; myoglobin [205]; serine [206]; urea [207,208]; viral diagnostics [209]; and other methods [190,193,210].

PEC biosensors were obtained using a nanosheet composite based on TiO_2_ and g-C_3_N_4_, which were able to detect glucose even at concentrations as low as 0.01 mM [211].

Using TiO_2_ samples decorated with gold nanoparticles, MnO_2,_ and g-C_3_N_4_ [203], not only glucose but also lactose was detected by PEC [176,192,197]. A more rapid test for glucose is from saliva [194] using amperometry from a PHT-TiO_2_ nanohybrid sample. The glucose was also detected by an impedimetric biosensor [195].

Minimally invasive procedures for sample collection are expected. Simple nano patches have been developed that can be applied to the skin surface for detection and diagnosis based on interstitial fluids exuded through skin pores. Additionally, the developed nano biosensors ought to be biodegradable to prevent any environmental harm [212,213].

Biosensors based on TiO_2_ NPs are successfully used not only in detection but also in cancer therapy or implantology.

#### 4.2.1. Cancer Detection

Oncological diseases appear suddenly and develop quickly, so an early diagnosis at the initial stage is extremely important. Aptasensors have been successfully applied in this field [177].

The most challenging goal of nanotechnology research in cancer therapy is the discovery of nanostructures for drug delivery and release in a manner that improves the therapeutic effect and reduces side effects [214].

In the biomedical field of TiO_2_ nanomaterials, only in 2023 were two important reviews published in the domain of drug delivery systems for cancer therapy, prevention, and treatment of infections, implants [211], as well as cancer diagnosis and its therapeutic systems [158]. In cancer treatment as well as in epilepsy, TiO_2_ NPs must be inserted directly into the diseased organ, so they must have appropriate shapes, like whiskers [215], hollow nanoparticles [216], pyramids [217], and rhombic [218]. A suitable/appropriate geometry leads to better incorporation of biomolecules into the sensor network, which facilitates the interaction between TiO_2_ molecules and biomarkers [219].

As previously mentioned, the use of TiO_2_ NPs can come with certain drawbacks, especially for human health. It was reported that titania nanoparticles can enter the placenta of pregnant women and affect the development of the fetus [220]. Additionally, TiO_2_ NPs can enter the bloodstream and agglomerate in the brain tissue, inducing neurotoxicity [221]. In the body, NPs can travel to different organs, such as the liver, spleen, heart, kidneys, etc., where they accumulate. Finally, due to their small size, these NPs can be easily inhaled, and in high doses, they can lead to respiratory diseases [222,223].

**Figure 14:**
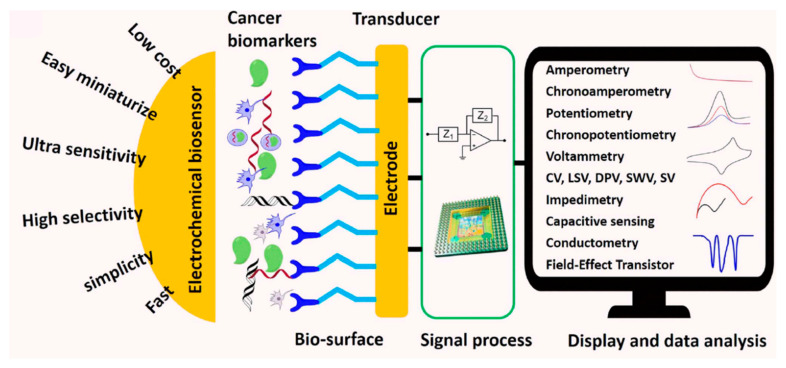
Benefits, components, and measurement approaches for electrochemical biosensors in cancer therapy. Reprinted from [224]. Copyright © 2020 by Cui, F., Zhou, Z., and Susan Zhou, H.

One of the usual biosensors used in the cancer field is the electrochemical one [158]. The benefits and the measurement approach are presented in Figure 14.

Since TiO_2_ NPs have light-controlled drug release properties, they can be used in radiotherapy for skin cancer or other therapy strategies. The most used sensors for cancer diagnosis, as mentioned above, are fluorescent biosensors. Kanehira et al. reported on TiO_2_-PEG nanoparticles and 5-aminolevulinic acid (ALA), which showed higher fluorescence durations and were used in the identification of cancer cells in the bladder [165].

The efficiency of the drugs is linked to delivery, which should be performed as close and as soon as possible to the infected area. For chronic deseases such as malignant cancers and epilepsy, the aim is to insert the NPs directly at the site of action.

#### 4.2.2. Implantology and Osteogeny

Titanium implants are well known and have been used for a long time. More recently, however, it has been found that titanium oxide could be an attractive alternative to titanium in bone, dental, or drug release implants. This is due to its low toxicity and high chemical stability, which prevent corrosion, but also to its higher flexibility (compared to titania) and high tensile strength [225]. Previous studies [226,227,228] have also reported on the antibacterial activity and osteogenic activity of titania implants.

The properties of titania, such as osseointegration, can be further enhanced by coupling it to different polymers with antioxidant properties (such as chitosan or chitosan-based polymers) [229]. Although already a highly stable material in an oxydizing environment, TiO_2_ surfaces can be further modified using UV irradiation to reduce inflammatory reactions and intracellular ROS production [230]. Titania nanotubes of 110 nm were shown to have excellent antioxidant and osteogenetic differentiation ability, much better than theirTi counterparts [231].

The use of titanium dioxide nanotubes is particularly attractive as they present a surface area nearly three times higher than that of Ti. This promotes higher cell adhesion, leading to improved osseointegration.

It was reported by Yu et al. [232] that, by using 100 nm diameter titania nanotube arrays (TNA), the implant was well integrated, with cell adhesion taking place at a good rate. This dimension of TNAs matches the topography of the hydroxyapatite in the collagen matrix, which explains the good match between the tissue and the implant.

## 5. Conclusions

This review covers the major results from recently published materials based on TiO_2_, highlighting improvements to its properties in various applications if it is used in 2D nanostructured forms (NP, NT, and NTAs), doped, or used in stacked films.

TiO_2_ as one of the more attractive multifunctional materials due to its low cost, non-toxicity, and chemical stability was surveyed in a wide range of applications such as(photo)catalysis, environmental applications (self-cleaning coatings), medicine (cancer therapy, implant fabrication/production, and bio-sensing), and human safety (gas sensing). Special attention was paid to the use of TiO_2_ as a photocatalyst for water treatment and self-cleaning or self-sterilizing coatings. In addition, the main differences, consisting of the possible advantages and disadvantages of using TiO_2_ as (nano)powder or as thin films, were presented.

The performance of TiO_2_ can be improved by tailoring the intrinsic properties through certain strategies: modifying the synthesis parameters (temperature, pH, and concentration), elemental doping (metal or non-metal), bandgap engineering, and creating heterojunctions.

Some of the main problems that have to be solved have been identified, which are as follows:

- Photoactivity loss of doped TiO_2_ during recycling and long-term storage. To this end, extending the activation domain to a larger range of visible light is imperative.
- Choosing the optimal type and concentration of dopant ions or carbon derivatives to engineer the bandgap energy of TiO_2_ is vital in order to attain photocatalytic and antimicrobial Vis activation.
- Regarding the use of TiO_2_ in the field of medicine, in-depth studies on the compatibility of the TiO_2_-based materials (implants and stents) with the components of the human body (tissues, cartilages, and bones) are needed.
- More intensive work is required to determine the TiO_2_ film’s cross-selectivity in the case of gas sensor applications.
- Preventing fluorescence extinction in the case of fluorescent biosensors.
- Oncological diseases appear suddenly and develop rapidly; therefore, early diagnosis in the initial stage is extremely important. In this respect, the development of rapid, safe, and precise diagnostic methods based on biosensors is expected.
- The development of portable sensors still remains a challenge.

*In conclusion, titanium dioxide was, is, and will be an intensively studied material that will make significant progress in the future in the photocatalysis and biomedical fields.*

## Figures and Tables

**Table 1:** The most recent reviews on TiO_2_.

No.	Year	Materials	Application	Main Results	Refs.
1	2018	Pristine TiO_2_, metal-assisted TiO_2_, organic–TiO_2_ composites, carbon–TiO_2_ composites, and MOX–TiO_2_ composites.	H_2_ gas sensor	The enhancement of the gas-sensing performance of TiO_2_-based materials through the synthesis route (doping, surface modification, and nano-fabrication) in terms of its effects on the properties and surface reaction mechanisms as a hydrogen sensor.	[5]
2	2019	TiO_2_ NPs	Self-cleaning, self-sterilizing, air purification, water disinfection, and antitumor activity	Explanation of the photocatalytic mechanism of TiO_2_ materials and their applicability in different fields; special attention was given to TiO_2_ NPs with antibacterial and self-cleaning properties to develop transparent coatings (super-hydrophobic and super-hydrophilic coatings) for windows in outdoor applications.	[6]
3	2020	TiO_2_/Pd TiO_2_/PdO	Visible light photocatalyst	Photocatalytic degradation of rhodamine B under visible light irradiation. The TiO_2_/PdO photocatalyst exhibits higher photocatalytic activity compared with TiO_2_/Pd, which makes it suitable for pollutant removal in water and wastewater treatments.	[7]
4	2020	TiO_2_ thin films, doped or in composites, and nanotubes.	Metal oxide gas sensor for H_2_, CO, NO, O_2_, NO_2_, C_3_H_4_, C_2_H_6_O, C_3_H_6_O, NH_3_, and C₆H₅CH₃	Effect of crystal structure, operating temperature, and doping with semiconductor oxides on the sensor properties of TiO_2_ thin films.	[8]
5	2021	Noble metals–TiO_2_ and carbon nanomaterials–TiO_2_ composites.	Water disinfection	Synthesis of TiO_2_ and TiO_2_-based composites as photocatalysts for water disinfection. The enhancement of the photocatalytic efficiency was achieved by adding noble metals and carbon materials to the TiO_2_ matrix. The impact of water properties on photocatalytic disinfection was also studied. Two important strategies to improve the photocatalytic efficiency of the materials were proposed: (1) the modification of the conduction band of the semiconductor to promote reactive species with longer lifetimes; (2) improving the bacteria and photocatalysts interactions.	[9]
6	2022	Hierarchical TiO_2_ nanostructure forms: 0D, 1D, 2D, and 3D.	Photocatalyst for oilfield-produced water (OPW) treatment	The possibility to enhance the photocatalytic activity for oilfield-produced water treatment (OPW) through the following pathways: energy band tailoring, obtaining and modifying TiO_2_ nanostructures by doping, and the development of photocatalytic membranes (PMs) based on TiO_2_.	[10]
7	2022	TiO_2_ nanohybrids (doping with metals, non-metals, co-doping, and graphene).	Food packaging	Food preservation and post-harvest loss mitigation applications. Nanotechnology has been explored as a scalable solution.	[1]
8	2022	Defective/reduced titanium dioxide (TiO_2−*x*_) and TiO_2−*x*_–carbon-based photocatalysts.	Photocatalytic CO_2_ reduction	Synthesis of TiO_2−*x*_ with different morphologies and TiO_2−*x*_-based materials. This study presents the changes in TiO_2_ properties according to the amount of oxygen vacancies and their performance as photocatalysts in CO_2_ reduction.	[11]
9	2022	TiO_2_ composites; metal- and non-metal TiO_2_-based photocatalysts.	Photocatalytic CO_2_ reduction	Synthesis methods to prepare photocatalysts for CO_2_ reduction into green products.	[12]
10	2022	TiO_2_ and other nanomaterial-based antimicrobial additives.	Functional paints and coatings	The incorporation of TiO_2_ nanoparticles in paint and coatings due to their interesting properties being used as additives with antibacterial properties, as inorganic binders to prevent organic binder photodegradation.	[13]
11	2023	Oxygen-deficient titanium oxide films with an average composition of TiO_2−δ._	Photocatalytic hydrogen production by water splitting	Explanation of the mechanism of photocatalytic hydrogen production by water splitting over TiO_2_. The introduction of oxygen defects into TiO_2_ through ion doping, the deposition of noble metals, and dye sensitization to improve the photocatalytic activity of TiO_2_-based materials.	[14]

**Table 2:** Materials based on TiO_2_ used in gas sensor applications.

Year	Material	Method	Substrate	Gas Detected	Main Results	Refs.
2018	Soot template TiO_2_ fractals	Chemical vapor deposition (CVD)	Deposited soot template (a layer of candle soot was deposited on Ti/Pt electrodes)	Acetone vapour	Novel structural design/diabetic concentrations in the breath.	[134]
2018	TiO_2_ films	Atomic layer deposition (ALD)	Alumina sensors and microscope slides	NH_3_ and CH_4_	Sensitivity toward NH_3_ varied with thickness.	[135]
2019	TiO_2_ NP	Bar coating	Alumina	H_2_S	Works at RT under UV.	[136]
2019	MgO:TiO_2_ thin films	Co-sputtering (confocal sputtering)	Pt/SiO_2_/Si	CH_4_	All the results showed that the dopant can improve the electrical performance and sensor properties.	[137]
2019	Nanostructured TiO_2_	Molecular layering (ML)	Al_2_O_3_ substrates with platinum electrodes	O_2_	ML-deposited TiO_2_ film shows good selectivity to oxygen.	[138]
2021	TiO_2_ thin film	Pulsed laser deposition (PLD)	-(100) silicon (Si/SiO_2_) -(100) SrTiO_3_ (STO) -polycrystalline Al_2_O_3_	H_2_S	The surface of PLD TiO_2_ film on Si/SiO_2_ has a higher roughness than that on the STO substrate.	[139]
Composite heterostructure						
2018	p-copper oxide thin film/n-TiO_2_Nts heterostructure	Anodization/ oxidation	Ti foil	H_2_, ethanol, acetone, chloroform, and NO_2_	The improvement in the sensing properties is attributed to the heterojunction between the CuO thin film and the TiO_2_ NTs.	[140]
2018	TiO_2_ + SnO_2_ NPs	SG+	Glass	NH_3_	The improvement in the sensor performance is due to the increased active surface area and the efficient electron–hole charge separation and transfer.	[141]
2019	Pd/Al_2_O_3_/TiO_2_ thin film heterostructure	Atomic layer deposition (ALD)	Quartz	H_2_	Development of flexible gas sensors.	[142]
2019	TiO_2_/perovskite heterojunctions	Electrochemical method, Sol–gel	Ti foil	CO	The heterojunction is more sensitive than a single film and could operate at lower temperatures.	[143]
2019	TiO_2_ film/carboxyl PP film/MWCNTs	ALD/CCVD	Si/SiO_2_	CO, H_2_, and NH_3_	Better sensor response, lower detection limit, and lower operation temperature against H_2_ compared to the separate sensors.	[144]
2019	Polyaniline (PANI)/TiO_2_ core–shell nanofibers	In situ chemical polymerization on electrospun TiO_2_ nanofibers	Glass	NH_3_	Under UV at RT at different humidity levels.	[145]
2019	TiO_2_/SnO_2_ and TiO_2_/CuO thin film nano-heterostructures	Reactive magnetron sputtering	Metallic target and a-SiO_2_	NO_2_	The response of TiO_2_/SnO_2_ to NO_2_ at 150 °C is double than in the case of pure SnO_2._	[146]
2019	ZnO (n-type)-TiO_2_ (n-type)-PANI (p-type) Micro/nanoballs	Chemical deposition technique	Glass	LPG, NO_2_, acetone, NH_3_, and CO_2_	Very good selectivity.	[130]
2020	TiO_2_ decorated with Au NPs	Irradiation with laser	Si and interdigital electrodes	Volatile organic compounds	Very selective and stable over time; works at RT.	[147]
2022	Pd doped CoTiO_3_/TiO_2_ (Pd-CTT)	HT	Interdigital electrodes	Benzene	Works at RT; good resistance to humidity interference.	[148]

## Data Availability

The data presented in this study are available on request from the corresponding author.

## Abbreviations

List of acronyms (in alphabetical order)

ALA	5-aminolevulinic acid
ALD	atomic layer deposition
AFM	atomic force microscopy
BBB	blood–brain barrier
C₆H₅CH₃	toluene
C6H6	benzene
CCVD	catalytic chemical vapour deposition
DNA	deoxyribonucleic acid
DSSC	Dye-sensitized solar cell
*E. coli*	*Escherichia coli*
Eg	energy bandgap
Ec	conduction band
E_F_	Fermi energy level
Ev	valence band
ENX	enoxacin
ES	Efros–Shklovskii
FET	field-effect transistor
GBs	grain boundaries
H_2_O_2_	hydrogen peroxide
HPV	Human Papillomavirus
HT	hydrothermal
IDEs	interdigital electrodes
IPA	isopropyl alcohol
I–V	current-voltage
ML	molecular layering method
MOS	metal–oxide–semiconductor
MOX	metal oxide
MWCNTs	multi-walled carbon nanotubes
NIR	near infrared
NPs	nanoparticles
NTs	nanotubes (TNT titania nanotubes)
NTAs	nanotubes arrays
OPW	oilfield-produced water
PANI	polyaniline
PDD	photodynamic diagnosis
PEC	(photo)electrochemical biosensors
PEG	polyethylene glycol
PEI	polyethyleneimine
PHT	polyhexahydrotriazine
PL	photoluminescence
PLD	pulsed laser deposition
PPy	polypyrrole
rGO	reduced graphene oxide
RMS	root mean square roughness
RNA	ribonucleic acid
ROS	reactive oxygen species
RT	room temperature
SEM	scanning electron microscopy
SG	sol–gel
SP	spray pyrolysis
SPR	plasmon resonance biosensor
STO	strontium titanate (SrTiO_3_)
TCO	transparent conductive oxide
TEM	transmission electron microscopy
TNA	titanium dioxide nanotube arrays
UV	ultraviolet
Vis	visible
VOCs	volatile organic components
VRH	variable range hopping
XRD	X-ray diffraction

## References

[1] Kodithuwakku P., Jayasundara D.R., Munaweera I., Jayasinghe R., Thoradeniya T., Weerasekera M., Ajayan P.M., Kottegoda N. (2022). A Review on Recent Developments in Structural Modification of TiO_2_ for Food Packaging Applications. Prog. Solid State Chem..

[2] EUR-Lex. https://eur-lex.europa.eu/eli/reg/2022/63/oj.

[3] Rashid M.M., Forte Tavčer P., Tomšič B. (2021). Influence of Titanium Dioxide Nanoparticles on Human Health and the Environment. Nanomaterials.

[4] Zaleska A. (2008). Doped-TiO_2_: A Review. Recent Pat. Eng..

[5] Li Z., Yao Z., Haidry A.A., Plecenik T., Xie L., Sun L., Fatima Q. (2018). Resistive-Type Hydrogen Gas Sensor Based on TiO_2_: A Review. Int. J. Hydrogen Energy.

[6] Haider A.J., Jameel Z.N., Al-Hussaini I.H.M. (2019). Review on: Titanium Dioxide Applications. Energy Procedia.

[7] Yan J., Li X., Jin B., Zeng M., Peng R. (2020). Synthesis of TiO_2_/Pd and TiO_2_/PdO Hollow Spheres and Their Visible Light Photocatalytic Activity. Int. J. Photoenergy.

[8] Malallah Rzaij J., Mohsen Abass A. (2020). Review on: TiO_2_ Thin Film as a Metal Oxide Gas Sensor. J. Chem. Rev..

[9] He J., Kumar A., Khan M., Lo I.M.C. (2021). Critical Review of Photocatalytic Disinfection of Bacteria: From Noble Metals- and Carbon Nanomaterials-TiO_2_ Composites to Challenges of Water Characteristics and Strategic Solutions. Sci. Total Environ..

[10] Dharma H.N.C., Jaafar J., Widiastuti N., Matsuyama H., Rajabsadeh S., Othman M.H.D., Rahman M.A., Jafri N.N.M., Suhaimin N.S., Nasir A.M. (2022). A Review of Titanium Dioxide (TiO_2_)-Based Photocatalyst for Oilfield-Produced Water Treatment. Membranes.

[11] Wang J., Guo R., Bi Z., Chen X., Hu X., Pan W. (2022). A Review on TiO_2−x_-Based Materials for Photocatalytic CO_2_ Reduction. Nanoscale.

[12] Rehman Z.U., Bilal M., Hou J., Butt F.K., Ahmad J., Ali S., Hussain A. (2022). Photocatalytic CO_2_ Reduction Using TiO_2_-Based Photocatalysts and TiO_2_ Z-Scheme Heterojunction Composites: A Review. Molecules.

[13] Chen M.C., Koh P.W., Ponnusamy V.K., Lee S.L. (2022). Titanium Dioxide and Other Nanomaterials Based Antimicrobial Additives in Functional Paints and Coatings: Review. Prog. Org. Coat..

[14] Chen Y., Fu X., Peng Z. (2023). A Review on Oxygen-Deficient Titanium Oxide for Photocatalytic Hydrogen Production. Metals.

[15] Reyes-Coronado D., Rodríguez-Gattorno G., Espinosa-Pesqueira M.E., Cab C., de Coss R., Oskam G. (2008). Phase-Pure TiO_2_ Nanoparticles: Anatase, Brookite and Rutile. Nanotechnology.

[16] Lance R. (2018). Optical Analysis of Titania: Band Gaps of Brookite, Rutile and Anatase.

[17] Kaur A., Umar A., Kansal S.K. (2015). Sunlight-Driven Photocatalytic Degradation of Non-Steroidal Anti-Inflammatory Drug Based on TiO_2_ Quantum Dots. J. Colloid Interface Sci..

[18] Gong Z., Yang N., Chen Z., Jiang B., Sun Y., Yang X., Zhang L. (2020). Fabrication of Meshes with Inverse Wettability Based on the TiO_2_ Nanowires for Continuous Oil/Water Separation. Chem. Eng. J..

[19] Zhang L., Bai H., Liu L., Sun D.D. (2018). Dimension Induced Intrinsic Physio-Electrical Effects of Nanostructured TiO_2_ on Its Antibacterial Properties. Chem. Eng. J..

[20] Cho K., Lee S., Kim H., Kim H.-E., Son A., Kim E., Li M., Qiang Z., Hong S.W. (2019). Effects of Reactive Oxidants Generation and Capacitance on Photoelectrochemical Water Disinfection with Self-Doped Titanium Dioxide Nanotube Arrays. Appl. Catal. B Environ..

[21] Şennik E., Alev O., Öztürk Z.Z. (2016). The Effect of Pd on the H_2_ and VOC Sensing Properties of TiO_2_ Nanorods. Sens. Actuators B Chem..

[22] Lan K., Liu Y., Zhang W., Liu Y., Elzatahry A., Wang R., Xia Y., Al-Dhayan D., Zheng N., Zhao D. (2018). Uniform Ordered Two-Dimensional Mesoporous TiO_2_ Nanosheets from Hydrothermal-Induced Solvent-Confined Monomicelle Assembly. J. Am. Chem. Soc..

[23] Gopal N.O., Basha M.H. (2018). TiO_2_ Nano-Flakes with High Activity Obtained from Phosphorus Doped TiO_2_ Nanoparticles by Hydrothermal Method. Ceram. Int..

[24] Tang H., Zhang D., Tang G., Ji X., Li C., Yan X., Wu Q. (2014). Low Temperature Synthesis and Photocatalytic Properties of Mesoporous TiO_2_ Nanospheres. J. Alloys Compd..

[25] Lu S., Yang S., Hu X., Liang Z., Guo Y., Xue Y., Cui H., Tian J. (2019). Fabrication of TiO_2_ Nanoflowers with Bronze (TiO_2_(B))/Anatase Heterophase Junctions for Efficient Photocatalytic Hydrogen Production. Int. J. Hydrogen Energy.

[26] Kondamareddy K.K., Neena D., Lu D., Peng T., Lopez M.A.M., Wang C., Yu Z., Cheng N., Fu D.J., Zhao X.Z. (2018). Ultra-Trace (Parts per Million-Ppm) W^6+^ Dopant Ions Induced Anatase to Rutile Transition (ART) of Phase Pure Anatase TiO_2_ Nanoparticles for Highly Efficient Visible Light-Active Photocatalytic Degradation of Organic Pollutants. Appl. Surf. Sci..

[27] Zhu Z., Wu S., Long Y., Zhang L., Xue X., Yin Y., Xu B. (2021). Phase-Transition Kinetics of Silicon-Doped Titanium Dioxide Based on High-Temperature X-Ray-Diffraction Measurements. J. Solid State Chem..

[28] Zhang L., Luo X., Zhang J.-D., Long Y.-F., Xue X., Xu B.-J. (2021). Kinetic Study on the Crystal Transformation of Fe-Doped TiO_2_ via In Situ High-Temperature X-Ray Diffraction and Transmission Electron Microscopy. ACS Omega.

[29] Kayani Z.N., Intizar T., Riaz S., Naseem S. (2021). Antibacterial, magnetic and dielectric properties of nano-structured V doped TiO_2_ thin films deposited by dip coating technique. Mater. Chem. Phys..

[30] Park J.T., Kim D.J., Kim D.H., Kim J.H. (2017). A Facile Graft Polymerization Approach to n-Doped TiO_2_ Heterostructures with Enhanced Visible-Light Photocatalytic Activity. Mater. Lett..

[31] Elmehasseb I., Kandil S., Elgendy K. (2020). Advanced Visible-Light Applications Utilizing Modified Zn-Doped TiO_2_ Nanoparticles via Non-Metal in Situ Dual Doping for Wastewater Detoxification. Optik.

[32] Sotelo-Vazquez C., Noor N., Kafizas A., Quesada-Cabrera R., Scanlon D.O., Taylor A., Durrant J.R., Parkin I.P. (2015). Multifunctional p-Doped TiO_2_ Films: A New Approach to Self-Cleaning, Transparent Conducting Oxide Materials. Chem. Mater..

[33] Asrafuzzaman F.N.U., Amin K.F., Gafur M.A., Gulshan F. (2023). Mangifera Indica Mediated Biogenic Synthesis of Undoped and Doped TiO_2_ Nanoparticles and Evaluation of Their Structural, Morphological, and Photocatalytic Properties. Results Mater..

[34] Avilés-García O., Espino-Valencia J., Romero-Romero R., Rico-Cerda J., Arroyo-Albiter M., Solís-Casados D., Natividad-Rangel R. (2018). Enhanced Photocatalytic Activity of Titania by Co-Doping with Mo and W. Catalysts.

[35] Wang Y., He Y., Lai Q., Fan M. (2014). Review of the Progress in Preparing Nano TiO_2_: An Important Environmental Engineering Material. J. Environ. Sci..

[36] Wang X., Li Z., Shi J., Yu Y. (2014). One-Dimensional Titanium Dioxide Nanomaterials: Nanowires, Nanorods, and Nanobelts. Chem. Rev..

[37] Bhandarkar S.A., Prathvi, Kompa A., Murari M.S., Kekuda D., Mohan R.K. (2021). Investigation of Structural and Optical Properties of Spin Coated TiO_2_:Mn Thin Films. Opt. Mater..

[38] Anitha V.C., Banerjee A.N., Joo S.W. (2015). Recent Developments in TiO_2_ as n- and p-Type Transparent Semiconductors: Synthesis, Modification, Properties, and Energy-Related Applications. J. Mater. Sci..

[39] Youssef A.M., Yakout S.M., Mousa S.M. (2023). High Relative Permittivity and Excellent Dye Photo-Elimination: Pure and (Zr^4+^, Y^3+^, Sb^5+^) Multi-Doped Anatase TiO_2_ Structure. Opt. Mater..

[40] Carp O. (2004). Photoinduced Reactivity of Titanium Dioxide. Prog. Solid State Chem..

[41] Vasu K., Sreedhara M.B., Ghatak J., Rao C.N.R. (2016). Atomic Layer Deposition of p-Type Epitaxial Thin Films of Undoped and n-Doped Anatase TiO_2_. ACS Appl. Mater. Interfaces.

[42] Yao W., Luo C., Wu J.B., Hou G. (2022). Degradation of Acid Yellow 36 Azo Dye from Textile Wastewater Using Vanadium-Doped TiO_2_ Photonanocatalyst. Int. J. Electrochem. Sci..

[43] Peighambardoust N.S., Khameneh Asl S., Mohammadpour R., Asl S.K. (2018). Band-Gap Narrowing and Electrochemical Properties in n-Doped and Reduced Anodic TiO_2_ Nanotube Arrays. Electrochim. Acta.

[44] Sayed M., Arooj A., Shah N.S., Khan J.A., Shah L.A., Rehman F., Arandiyan H., Khan A.M., Khan A.R. (2018). Narrowing the Band Gap of TiO_2_ by Co-Doping with Mn^2+^ and Co^2+^ for Efficient Photocatalytic Degradation of Enoxacin and Its Additional Peroxidase like Activity: A Mechanistic Approach. J. Mol. Liq..

[45] Joshi P., Tiwari S., Punia K., Kumar S. (2022). Defect Mediated Mechanism in Greenly Synthesized Undoped, Al^+3^, Cu^+2^ and Zn^+2^ Doped TiO_2_ Nanoparticles for Tailoring Bandgap, Luminescence, Magnetic and Electrical Properties. Opt. Mater..

[46] Yan H., Wang R., Liu R., Xu T., Sun J., Liu L., Wang J. (2021). Recyclable and Reusable Direct Z-Scheme Heterojunction CeO_2_/TiO_2_ Nanotube Arrays for Photocatalytic Water Disinfection. Appl. Catal. B Environ..

[47] Roose B., Pathak S., Steiner U. (2015). Doping of TiO_2_ for Sensitized Solar Cells. Chem. Soc. Rev..

[48] Lettieri S., Pavone M., Fioravanti A., Santamaria Amato L., Maddalena P. (2021). Charge Carrier Processes and Optical Properties in TiO_2_ and TiO_2_-Based Heterojunction Photocatalysts: A Review. Materials.

[49] Nowotny M.K., Bak T., Nowotny J. (2006). Electrical Properties and Defect Chemistry of TiO_2_ Single Crystal. I. Electrical Conductivity. J. Phys. Chem. B.

[50] Nowotny J., Bak T., Nowotny M., Sheppard L. (2007). Titanium Dioxide for Solar-Hydrogen II. Defect Chemistry. Int. J. Hydrogen Energy.

[51] Bi Z., Li K., Jiang C., Zhang J., Ma S., Alberto C., Sun M., Bu Y., Barati M., Ren S. (2022). New Insights into the Traditional Charge Compensation Theory: Amphoteric Behavior of TiO_2_ under the Guidance of Supply–Demand Relationship. ACS Omega.

[52] Chen X., Mao S.S. (2007). Titanium Dioxide Nanomaterials: Synthesis, Properties, Modifications, and Applications. Chem. Rev..

[53] Morgan B.J., Watson G.W. (2010). Intrinsic n-Type Defect Formation in TiO_2_: A Comparison of Rutile and Anatase from GGA+ U Calculations. J. Phys. Chem. C.

[54] Nakamura I., Negishi N., Kutsuna S., Ihara T., Sugihara S., Takeuchi K. (2000). Role of Oxygen Vacancy in the Plasma-Treated TiO_2_ Photocatalyst with Visible Light Activity for NO Removal. J. Mol. Catal. A Chem..

[55] Pan X., Yang M.-Q., Fu X., Zhang N., Xu Y.-J. (2013). Defective TiO_2_ with Oxygen Vacancies: Synthesis, Properties and Photocatalytic Applications. Nanoscale.

[56] Simeonov S., Szekeres A., Covei M., Spassov D., Kitin G., Predoana L., Calderon-Moreno J.M., Nicolescu M., Preda S., Stroescu H. (2020). Inter-Trap Tunneling in Vanadium Doped TiO_2_ Sol-Gel Films. Mater. Res. Bull..

[57] Wang S., Pan L., Song J.-J., Mi W., Zou J.-J., Wang L., Zhang X. (2015). Titanium-Defected Undoped Anatase TiO_2_ with p-Type Conductivity, Room-Temperature Ferromagnetism, and Remarkable Photocatalytic Performance. J. Am. Chem. Soc..

[58] Cohen M.J., Paul M.D., Miller D.L., Waldrop J.R., Harris J.S. (1980). Schottky Barrier Behavior in Polycrystal GaAs. J. Vac. Sci. Technol..

[59] McPherson J.W., Collis W., Stefanakos E., Safavi A., Abul-Fadl A. (1980). Band Bending and Passivation Studies of GaAs Grain Boundaries. J. Electrochem. Soc..

[60] Möller H.J., Strunk H.P., Werner J.H., Möller H.J., Strunk H.P., Werner J.H. (1989). Polycrystalline Semiconductors.

[61] Greuter F., Blatter G. (1990). Electrical Properties of Grain Boundaries in Polycrystalline Compound Semiconductors. Semicond. Sci. Technol..

[62] Rathee D., Arya S.K., Kumar M. (2011). Analysis of TiO_2_ for Microelectronic Applications: Effect of Deposition Methods on Their Electrical Properties. Front. Optoelectron. China.

[63] Simeonov S., Kafedjiiska E., Szekeres A., Parlog C., Gartaer M. (2005). Electrical Characterization of MIS Structures with Sol-Gel TiO_2_(La) Dielectric Films. J. Optoelectron. Adv. Mater..

[64] Kumar A., Mondal S., Rao K.S.R.K. (2015). DLTS Analysis of Amphoteric Interface Defects in High-TiO_2_ MOS Structures Prepared by Sol-Gel Spin-Coating. AIP Adv..

[65] Gyanan, Mondal S., Kumar A. (2016). Tunable Dielectric Properties of TiO_2_ Thin Film Based MOS Systems for Application in Microelectronics. Superlattices Microstruct..

[66] Kumar A., Mondal S., Koteswara Rao K.S.R. (2016). Critical Investigation of High Performance Spin-Coated High-κ Titania Thin Films Based MOS Capacitor. J. Mater. Sci. Mater. Electron..

[67] Mech B.C., Kumar J. (2017). Effect of High-k Dielectric on the Performance of Si, InAs and CNT FET. Micro Nano Lett..

[68] Rothschild A., Komem Y., Levakov A., Ashkenasy N., Shapira Y. (2003). Electronic and Transport Properties of Reduced and Oxidized Nanocrystalline TiO_2_ Films. Appl. Phys. Lett..

[69] Mehdizadeh P., Tavangar Z., Shabani N., Hamadanian M. (2020). Visible Light Activity of Nitrogen-Doped TiO_2_ by Sol-Gel Method Using Various Nitrogen Sources. J. Nanostruct..

[70] Das S., Liu D., Park J.B., Hahn Y.B. (2013). Metal-Ion Doped p-Type TiO_2_ Thin Films and Their Applications for Heterojunction Devices. J. Alloys Compd..

[71] Yildiz A., Lisesivdin S.B., Kasap M., Mardare D. (2008). Electrical Properties of TiO_2_ Thin Films. J. Non. Cryst. Solids.

[72] Kneiß M., Jenderka M., Brachwitz K., Lorenz M., Grundmann M. (2014). Modeling the Electrical Transport in Epitaxial Undoped and Ni-, Cr-, and W-Doped TiO_2_ Anatase Thin Films. Appl. Phys. Lett..

[73] Mott N.F. (1968). Conduction in Glasses Containing Transition Metal Ions. J. Non. Cryst. Solids.

[74] Shklovskii B.I., Efros A.L. (1984). Electronic Properties of Doped Semiconductors.

[75] Zhao Y.L., Lv W.M., Liu Z.Q., Zeng S.W., Motapothula M., Dhar S., Ariando, Wang Q., Venkatesan T. (2012). Variable Range Hopping in TiO_2_ Insulating Layers for Oxide Electronic Devices. AIP Adv..

[76] Monteagudo J.M., Durán A., Martín I.S., Acevedo A.M. (2017). A Novel Combined Solar Pasteurizer/TiO_2_ Continuous-Flow Reactor for Decontamination and Disinfection of Drinking Water. Chemosphere.

[77] Nyangaresi P.O., Qin Y., Chen G., Zhang B., Lu Y., Shen L. (2019). Comparison of UV-LED Photolytic and UV-LED/TiO_2_ Photocatalytic Disinfection for Escherichia Coli in Water. Catal. Today.

[78] De Pasquale I., Lo Porto C., Dell’Edera M., Curri M.L., Comparelli R. (2021). TiO_2_-Based Nanomaterials Assisted Photocatalytic Treatment for Virus Inactivation: Perspectives and Applications. Curr. Opin. Chem. Eng..

[79] Khaiboullina S., Uppal T., Dhabarde N., Subramanian V.R., Verma S.C. (2020). Inactivation of Human Coronavirus by Titania Nanoparticle Coatings and UVC Radiation: Throwing Light on SARS-CoV-2. Viruses.

[80] Wu M.-C., Lin T.-H., Hsu K.-H., Hsu J.-F. (2019). Photo-Induced Disinfection Property and Photocatalytic Activity Based on the Synergistic Catalytic Technique of Ag Doped TiO_2_ Nanofibers. Appl. Surf. Sci..

[81] Gadgil D.J., Shetty Kodialbail V. (2021). Suspended and Polycaprolactone Immobilized Ag@TiO_2_/Polyaniline Nanocomposites for Water Disinfection and Endotoxin Degradation by Visible and Solar Light-Mediated Photocatalysis. Environ. Sci. Pollut. Res..

[82] Torres-Limiñana J., Feregrino-Pérez A.A., Vega-González M., Escobar-Alarcón L., Cervantes-Chávez J.A., Esquivel K. (2022). Green Synthesis via *Eucalyptus globulus* L. Extract of Ag-TiO_2_ Catalyst: Antimicrobial Activity Evaluation toward Water Disinfection Process. Nanomaterials.

[83] Miao Y., Xu X., Liu K., Wang N. (2017). Preparation of Novel Cu/TiO_2_ Mischcrystal Composites and Antibacterial Activities for Escherichia Coli under Visible Light. Ceram. Int..

[84] Pablos C., Marugán J., van Grieken R., Dunlop P., Hamilton J., Dionysiou D., Byrne J. (2017). Electrochemical Enhancement of Photocatalytic Disinfection on Aligned TiO_2_ and Nitrogen Doped TiO_2_ Nanotubes. Molecules.

[85] Makropoulou T., Panagiotopoulou P., Venieri D. (2018). n-doped TiO_2_ Photocatalysts for Bacterial Inactivation in Water. J. Chem. Technol. Biotechnol..

[86] Levchuk I., Homola T., Moreno-Andrés J., Rueda-Márquez J.J., Dzik P., Moríñigo M.Á., Sillanpää M., Manzano M.A., Vahala R. (2019). Solar Photocatalytic Disinfection Using Ink-Jet Printed Composite TiO_2_/SiO_2_ Thin Films on Flexible Substrate: Applicability to Drinking and Marine Water. Sol. Energy.

[87] Keeley M., Kisslinger K., Adamson C., Furlan P.Y. (2021). Magnetically Recoverable and Reusable Titanium Dioxide Nanocomposite for Water Disinfection. J. Mar. Sci. Eng..

[88] Zeng X., Wang Z., Wang G., Gengenbach T.R., McCarthy D.T., Deletic A., Yu J., Zhang X. (2017). Highly Dispersed TiO_2_ Nanocrystals and WO_3_ Nanorods on Reduced Graphene Oxide: Z-Scheme Photocatalysis System for Accelerated Photocatalytic Water Disinfection. Appl. Catal. B Environ..

[89] Berberidou C., Kyzas G.Z., Paspaltsis I., Sklaviadis T., Poulios I. (2019). Photocatalytic Disinfection and Purification of Water Employing Reduced Graphene Oxide/TiO_2_ Composites. J. Chem. Technol. Biotechnol..

[90] Dey B., Bulou S., Ravisy W., Gautier N., Richard-Plouet M., Granier A., Choquet P. (2022). Low-Temperature Deposition of Self-Cleaning Anatase TiO_2_ Coatings on Polymer Glazing via Sequential Continuous and Pulsed PECVD. Surf. Coat. Technol..

[91] Covei M., Bogatu C., Gheorghita S., Duta A., Stroescu H., Nicolescu M., Calderon-Moreno J.M., Atkinson I., Bratan V., Gartner M. (2023). Influence of the Deposition Parameters on the Properties of TiO_2_ Thin Films on Spherical Substrates. Materials.

[92] Singh J., Sahu K., Pandey A., Kumar M., Ghosh T., Satpati B., Som T., Varma S., Avasthi D.K., Mohapatra S. (2017). Atom Beam Sputtered Ag-TiO_2_ Plasmonic Nanocomposite Thin Films for Photocatalytic Applications. Appl. Surf. Sci..

[93] Scarisoreanu M., Ilie A.G., Goncearenco E., Banici A.M., Morjan I.P., Dutu E., Tanasa E., Fort I., Stan M., Mihailescu C.N. (2020). Ag, Au and Pt Decorated TiO_2_ Biocompatible Nanospheres for UV&Vis Photocatalytic Water Treatment. Appl. Surf. Sci..

[94] Cao T., Dong W., Liang Y., Bao Q., Xu C., Bai M., Luo T., Gu X. (2023). A Simple Solvothermal Preparation of Mg-Doped Anatase TiO_2_ and Its Self-Cleaning Application. Sol. Energy.

[95] Mahanta U., Khandelwal M., Deshpande A.S. (2022). TiO_2_@SiO_2_ Nanoparticles for Methylene Blue Removal and Photocatalytic Degradation under Natural Sunlight and Low-Power UV Light. Appl. Surf. Sci..

[96] Akhter P., Nawaz S., Shafiq I., Nazir A., Shafique S., Jamil F., Park Y.-K., Hussain M. (2023). Efficient Visible Light Assisted Photocatalysis Using ZnO/TiO_2_ Nanocomposites. Mol. Catal..

[97] Bouziani A., Park J., Ozturk A. (2020). Synthesis of α-Fe_2_O_3_/TiO_2_ Heterogeneous Composites by the Sol-Gel Process and Their Photocatalytic Activity. J. Photochem. Photobiol. A Chem..

[98] Wang K., Ruan J., Song H., Zhang J., Wo Y., Guo S., Cui D. (2010). Biocompatibility of Graphene Oxide. Nanoscale Res. Lett..

[99] Ma Y., Zhi L. (2019). Graphene-based Transparent Conductive Films: Material Systems, Preparation and Applications. Small Methods.

[100] Rauwel P., Galeckas A., Ducroquet F., Rauwel E. (2019). Selective Photocurrent Generation in HfO_2_ and Carbon Nanotube Hybrid Nanocomposites under Ultra-Violet and Visible Photoexcitations. Mater. Lett..

[101] Koli V.B., Dhodamani A.G., Raut A.V., Thorat N.D., Pawar S.H., Delekar S.D. (2016). Visible Light Photo-Induced Antibacterial Activity of TiO_2_-MWCNTs Nanocomposites with Varying the Contents of MWCNTs. J. Photochem. Photobiol. A Chem..

[102] Tian H., Shen K., Hu X., Qiao L., Zheng W. (2017). N, S Co-Doped Graphene Quantum Dots-Graphene-TiO_2_ Nanotubes Composite with Enhanced Photocatalytic Activity. J. Alloys Compd..

[103] Afzal M.J., Pervaiz E., Farrukh S., Ahmed T., Bingxue Z., Yang M. (2019). Highly Integrated Nanocomposites of RGO/TiO_2_ Nanotubes for Enhanced Removal of Microbes from Water. Environ. Technol..

[104] Xu W., Xie W., Huang X., Chen X., Huang N., Wang X., Liu J. (2017). The Graphene Oxide and Chitosan Biopolymer Loads TiO_2_ for Antibacterial and Preservative Research. Food Chem..

[105] Raja A., Selvakumar K., Rajasekaran P., Arunpandian M., Ashokkumar S., Kaviyarasu K., Asath Bahadur S., Swaminathan M. (2019). Visible Active Reduced Graphene Oxide Loaded Titania for Photodecomposition of Ciprofloxacin and Its Antibacterial Activity. Colloids Surfaces A Physicochem. Eng. Asp..

[106] Gillespie P.N.O., Martsinovich N. (2019). Origin of Charge Trapping in TiO_2_/Reduced Graphene Oxide Photocatalytic Composites: Insights from Theory. ACS Appl. Mater. Interfaces.

[107] Giovannetti R., Rommozzi E., Zannotti M., D’Amato C.A. (2017). Recent Advances in Graphene Based TiO_2_ Nanocomposites (GTiO_2_Ns) for Photocatalytic Degradation of Synthetic Dyes. Catalysts.

[108] Sharma M., Behl K., Nigam S., Joshi M. (2018). TiO_2_-GO Nanocomposite for Photocatalysis and Environmental Applications: A Green Synthesis Approach. Vacuum.

[109] Covei M., Perniu D., Bogatu C., Duta A., Visa I. (2022). Photocatalytic Composite Thin Films with Controlled Optical Properties Based on TiO_2_, WO_3_ and RGO. Surfaces Interfaces.

[110] Covei M., Visa I., Duta A. (2023). Self-Cleaning Photocatalytic Ceramic Coatings. Advanced Ceramic Coatings.

[111] Lin N., Verma D., Saini N., Arbi R., Munir M., Jovic M., Turak A. (2021). Antiviral Nanoparticles for Sanitizing Surfaces: A Roadmap to Self-Sterilizing against COVID-19. Nano Today.

[112] Sadowski R., Wach A., Buchalska M., Kuśtrowski P., Macyk W. (2019). Photosensitized TiO_2_ Films on Polymers–Titania-Polymer Interactions and Visible Light Induced Photoactivity. Appl. Surf. Sci..

[113] Rtimi S., Giannakis S., Pulgarin C. (2017). Self-Sterilizing Sputtered Films for Applications in Hospital Facilities. Molecules.

[114] Rtimi S., Pulgarin C., Robyr M., Aybush A., Shelaev I., Gostev F., Nadtochenko V., Kiwi J. (2017). Insight into the Catalyst/Photocatalyst Microstructure Presenting the Same Composition but Leading to a Variance in Bacterial Reduction under Indoor Visible Light. Appl. Catal. B Environ..

[115] Rtimi S., Robyr M., Pulgarin C., Lavanchy J.C., Kiwi J. (2016). A New Perspective in the Use of FeO_x_-TiO_2_ Photocatalytic Films: Indole Degradation in the Absence of Fe-Leaching. J. Catal..

[116] Syafiq A., Vengadaesvaran B., Pandey A.K., Rahim N.A. (2018). Superhydrophilic Smart Coating for Self-Cleaning Application on Glass Substrate. J. Nanomater..

[117] Luna M., Delgado J., Gil M., Mosquera M. (2018). TiO_2_-SiO_2_ Coatings with a Low Content of AuNPs for Producing Self-Cleaning Building Materials. Nanomaterials.

[118] Bergamonti L., Predieri G., Paz Y., Fornasini L., Lottici P.P., Bondioli F. (2017). Enhanced Self-Cleaning Properties of N-Doped TiO_2_ Coating for Cultural Heritage. Microchem. J..

[119] Veziroglu S., Hwang J., Drewes J., Barg I., Shondo J., Strunskus T., Polonskyi O., Faupel F., Aktas O.C. (2020). PdO Nanoparticles Decorated TiO_2_ Film with Enhanced Photocatalytic and Self-Cleaning Properties. Mater. Today Chem..

[120] Padmanabhan N.T., John H. (2020). Titanium Dioxide Based Self-Cleaning Smart Surfaces: A Short Review. J. Environ. Chem. Eng..

[121] Kavitha M.K., Rolland L., Johnson L., John H., Jayaraj M.K. (2020). Visible Light Responsive Superhydrophilic TiO_2_/Reduced Graphene Oxide Coating by Vacuum-Assisted Filtration and Transfer Method for Self-Cleaning Application. Mater. Sci. Semicond. Process..

[122] Salarbashi D., Tafaghodi M., Bazzaz B.S.F. (2018). Soluble Soybean Polysaccharide/TiO_2_ Bionanocomposite Film for Food Application. Carbohydr. Polym..

[123] Dias V., Maciel H., Fraga M., Lobo A., Pessoa R., Marciano F. (2019). Atomic Layer Deposited TiO_2_ and Al_2_O_3_ Thin Films as Coatings for Aluminum Food Packaging Application. Materials.

[124] Athir N., Shah S.A.A., Shehzad F.K., Cheng J., Zhang J., Shi L. (2020). Rutile TiO_2_ Integrated Zwitterion Polyurethane Composite Films as an Efficient Photostable Food Packaging Material. React. Funct. Polym..

[125] Anaya-Esparza L.M., Ruvalcaba-Gómez J.M., Maytorena-Verdugo C.I., González-Silva N., Romero-Toledo R., Aguilera-Aguirre S., Pérez-Larios A., Montalvo-González E. (2020). Chitosan-TiO_2_: A Versatile Hybrid Composite. Materials.

[126] Abutalib M.M., Rajeh A. (2021). Enhanced Structural, Electrical, Mechanical Properties and Antibacterial Activity of Cs/PEO Doped Mixed Nanoparticles (Ag/TiO_2_) for Food Packaging Applications. Polym. Test..

[127] Mesgari M., Aalami A.H., Sahebkar A. (2021). Antimicrobial Activities of Chitosan/Titanium Dioxide Composites as a Biological Nanolayer for Food Preservation: A Review. Int. J. Biol. Macromol..

[128] Van Nguyen S., Lee B.-K. (2022). PVA/CNC/TiO_2_ Nanocomposite for Food-Packaging: Improved Mechanical, UV/Water Vapor Barrier, and Antimicrobial Properties. Carbohydr. Polym..

[129] Zhang W., Rhim J.-W. (2022). Titanium Dioxide (TiO_2_) for the Manufacture of Multifunctional Active Food Packaging Films. Food Packag. Shelf Life.

[130] Sonker R.K., Yadav B.C.C., Gupta V., Tomar M. (2019). Fabrication and Characterization of ZnO-TiO_2_-PANI (ZTP) Micro/Nanoballs for the Detection of Flammable and Toxic Gases. J. Hazard. Mater..

[131] Chesler P., Vladut C.M. (2020). Senzori Chimici Rezistivi.

[132] Decroly A., Krumpmann A., Debliquy M., Lahem D. (2016). Nanostructured TiO_2_ Layers for Photovoltaic and Gas Sensing Applications. Green Nanotechnology-Overview and Further Prospects.

[133] Chesler P., Hornoiu C., Bratan V., Munteanu C., Gartner M., Ionescu N.I. (2015). Carbon Monoxide Sensing Properties of TiO_2_. Rev. Roum. Chim..

[134] Sabri Y.M., Kandjani A.E., Rashid S.S.A.A.H., Harrison C.J., Ippolito S.J., Bhargava S.K. (2018). Soot Template TiO_2_ Fractals as a Photoactive Gas Sensor for Acetone Detection. Sens. Actuators B Chem..

[135] Wilson R.L., Simion C.E., Blackman C.S., Carmalt C.J., Stanoiu A., Di Maggio F., Covington J.A. (2018). The Effect of Film Thickness on the Gas Sensing Properties of Ultra-Thin TiO_2_ Films Deposited by Atomic Layer Deposition. Sensors.

[136] Chinh N.D., Kim C., Kim D. (2019). UV-Light-Activated H_2_S Gas Sensing by a TiO_2_ Nanoparticulate Thin Film at Room Temperature. J. Alloys Compd..

[137] Sertel B.C., Sonmez N.A., Kaya M.D., Ozcelik S. (2019). Development of MgO:TiO_2_ Thin Films for Gas Sensor Applications. Ceram. Int..

[138] Mokrushin A.S., Simonenko E.P., Simonenko N.P., Akkuleva K.T., Antipov V.V., Zaharova N.V., Malygin A.A., Bukunov K.A., Sevastyanov V.G., Kuznetsov N.T. (2019). Oxygen Detection Using Nanostructured TiO_2_ Thin Films Obtained by the Molecular Layering Method. Appl. Surf. Sci..

[139] Nagmani, Pravarthana D., Tyagi A., Jagadale T.C., Prellier W., Aswal D.K. (2021). Highly Sensitive and Selective H_2_S Gas Sensor Based on TiO_2_ Thin Films. Appl. Surf. Sci..

[140] Alev O., Şennik E., Öztürk Z.Z. (2018). Improved Gas Sensing Performance of p-Copper Oxide Thin Film/n-TiO_2_ Nanotubes Heterostructure. J. Alloys Compd..

[141] Patil S.M., Dhodamani A.G., Vanalakar S.A., Deshmukh S.P., Delekar S.D. (2018). Multi-Applicative Tetragonal TiO_2_/SnO_2_ Nanocomposites for Photocatalysis and Gas Sensing. J. Phys. Chem. Solids.

[142] Kim S.M., Kim H.J., Jung H.J., Park J.Y., Seok T.J., Choa Y.H., Park T.J., Lee S.W. (2019). High-Performance, Transparent Thin Film Hydrogen Gas Sensor Using 2D Electron Gas at Interface of Oxide Thin Film Heterostructure Grown by Atomic Layer Deposition. Adv. Funct. Mater..

[143] Hsu K.-C., Fang T.-H., Hsiao Y.-J., Wu P.-C. (2019). Response and Characteristics of TiO_2_/Perovskite Heterojunctions for CO Gas Sensors. J. Alloys Compd..

[144] Kaushik P., Eliáš M., Michalička J., Hegemann D., Pytlíček Z., Nečas D., Zajíčková L. (2019). Atomic Layer Deposition of Titanium Dioxide on Multi-Walled Carbon Nanotubes for Ammonia Gas Sensing. Surf. Coat. Technol..

[145] Seif A., Nikfarjam A., Ghassem H.H., Safe A.M., Nikfarjam A., Hajghassem H. (2019). UV Enhanced Ammonia Gas Sensing Properties of PANI/TiO_2_ Core-Shell Nanofibers. Sens. Actuators B Chem..

[146] Maziarz W. (2019). TiO_2_/SnO_2_ and TiO_2_/CuO Thin Film Nano-Heterostructures as Gas Sensors. Appl. Surf. Sci..

[147] Mintcheva N., Srinivasan P., Rayappan J.B.B., Kuchmizhak A.A., Gurbatov S., Kulinich S.A. (2020). Room-Temperature Gas Sensing of Laser-Modified Anatase TiO_2_ Decorated with Au Nanoparticles. Appl. Surf. Sci..

[148] Wang D., Zhang D., Mi Q. (2022). A High-Performance Room Temperature Benzene Gas Sensor Based on CoTiO_3_ Covered TiO_2_ Nanospheres Decorated with Pd Nanoparticles. Sens. Actuators B Chem..

[149] Duta M., Predoana L., Calderon-Moreno J.M., Preda S., Anastasescu M., Marin A., Dascalu I., Chesler P., Hornoiu C., Zaharescu M. (2016). Nb-Doped TiO_2_ Sol–Gel Films for CO Sensing Applications. Mater. Sci. Semicond. Process..

[150] Bairi V.G., Bourdo S.E., Sacre N., Nair D., Berry B.C., Biris A.S., Viswanathan T. (2015). Ammonia Gas Sensing Behavior of Tanninsulfonic Acid Doped Polyaniline-TiO_2_ Composite. Sensors.

[151] Pang Z., Yang Z., Chen Y., Zhang J., Wang Q., Huang F., Wei Q. (2016). A Room Temperature Ammonia Gas Sensor Based on Cellulose/TiO_2_/PANI Composite Nanofibers. Colloids Surfaces A Physicochem. Eng. Asp..

[152] Kumar A., Sanger A., Kumar A., Chandra R. (2016). Fast Response Ammonia Sensors Based on TiO_2_ and NiO Nanostructured Bilayer Thin Films. RSC Adv..

[153] Castillero P., Roales J., Lopes-Costa T., Sánchez-Valencia J.R., Barranco A., González-Elipe A.R., Pedrosa J.M. (2017). Optical Gas Sensing of Ammonia and Amines Based on Protonated Porphyrin/TiO_2_ Composite Thin Films. Sensors.

[154] Li X., Zhao Y., Wang X., Wang J., Gaskov A.M., Akbar S.A. (2016). Reduced Graphene Oxide (RGO) Decorated TiO_2_ Microspheres for Selective Room-Temperature Gas Sensors. Sens. Actuators B Chem..

[155] Boyadjiev S.I., Kéri O., Bárdos P., Firkala T., Gáber F., Nagy Z.K., Baji Z., Takács M., Szilágyi I.M. (2017). TiO_2_/ZnO and ZnO/TiO_2_Core/Shell Nanofibers Prepared by Electrospinning and Atomic Layer Deposition for Photocatalysis and Gas Sensing. Appl. Surf. Sci..

[156] Liu C., Tai H., Zhang P., Ye Z., Su Y., Jiang Y. (2017). Enhanced Ammonia-Sensing Properties of PANI-TiO_2_-Au Ternary Self-Assembly Nanocomposite Thin Film at Room Temperature. Sens. Actuators B Chem..

[157] Ye Z., Tai H., Guo R., Yuan Z., Liu C., Su Y., Chen Z., Jiang Y. (2017). Excellent Ammonia Sensing Performance of Gas Sensor Based on Graphene/Titanium Dioxide Hybrid with Improved Morphology. Appl. Surf. Sci..

[158] Pourmadadi M., Rajabzadeh-Khosroshahi M., Eshaghi M.M., Rahmani E., Motasadizadeh H., Arshad R., Rahdar A., Pandey S. (2023). TiO_2_-Based Nanocomposites for Cancer Diagnosis and Therapy: A Comprehensive Review. J. Drug Deliv. Sci. Technol..

[159] Nadzirah S., Gopinath S.C.B., Parmin N.A., Hamzah A.A., Mohamed M.A., Chang E.Y., Dee C.F. (2022). State-of-the-Art on Functional Titanium Dioxide-Integrated Nano-Hybrids in Electrical Biosensors. Crit. Rev. Anal. Chem..

[160] Brainina K.Z. (1995). Sensors and Sample Preparation in Stripping Voltammetry. Anal. Chim. Acta.

[161] Mathew S., Kumar Prasad A., Benoy T., Rakesh P.P., Hari M., Libish T.M., Radhakrishnan P., Nampoori V.P.N., Vallabhan C.P.G. (2012). UV-Visible Photoluminescence of TiO_2_ Nanoparticles Prepared by Hydrothermal Method. J. Fluoresc..

[162] Ariño C., Serrano N., Díaz-Cruz J.M., Esteban M. (2017). Voltammetric Determination of Metal Ions beyond Mercury Electrodes. A Review. Anal. Chim. Acta.

[163] Brainina K.Z. (1971). Film Stripping Voltammetry. Talanta.

[164] Brainina K.Z. (1981). Inverse Voltammetry (Stripping Analysis) in the Investigation of Biologically Important Compounds. J. Electroanal. Chem. Interfacial Electrochem..

[165] Kanehira K., Yano Y., Hasumi H., Fukuhara H., Inoue K., Hanazaki K., Yao M. (2019). Fluorescence Enhancement Effect of TiO_2_ Nanoparticles and Application for Photodynamic Diagnosis. Int. J. Mol. Sci..

[166] Adzhri R., Arshad M.M., Gopinath S.C., Ruslinda A.R., Fathil M.F., Ibau C., Nuzaihan M. (2017). Enhanced Sensitivity Mediated Ambipolar Conduction with p-Type TiO_2_ Anatase Transducer for Biomarker Capturing. Sens. Actuators A Phys..

[167] Azizah N., Hashim U., Nadzirah S., Ruslinda A.R. (2014). Rapid and Sensitive Strategy for Human Papillomavirus (HPV) Detection Using a Gene-Based DNA Nanobiosensor. Proceedings of the 2014 IEEE Conference on Biomedical Engineering and Sciences (IECBES).

[168] Xu C., Thakur A., Li Z., Yang T., Zhao C., Li Y., Lee Y., Wu C.-M.L. (2021). Determination of Glioma Cells’ Malignancy and Their Response to TMZ via Detecting Exosomal BIGH3 by a TiO_2_-CTFE-AuNIs Plasmonic Biosensor. Chem. Eng. J..

[169] Yan S.R., Foroughi M.M., Safaei M., Jahani S., Ebrahimpour N., Borhani F., Rezaei Zade Baravati N., Aramesh-Boroujeni Z., Foong L.K. (2020). A Review: Recent Advances in Ultrasensitive and Highly Specific Recognition Aptasensors with Various Detection Strategies. Int. J. Biol. Macromol..

[170] Liu L.S., Wang F., Ge Y., Lo P.K. (2021). Recent Developments in Aptasensors for Diagnostic Applications. ACS Appl. Mater. Interfaces.

[171] Wang L., Chen X., Wang X., Han X., Liu S., Zhao C. (2011). Electrochemical Synthesis of Gold Nanostructure Modified Electrode and Its Development in Electrochemical DNA Biosensor. Biosens. Bioelectron..

[172] Skotadis E., Voutyras K., Chatzipetrou M., Tsekenis G., Patsiouras L., Madianos L., Chatzandroulis S., Zergioti I., Tsoukalas D. (2016). Label-Free DNA Biosensor Based on Resistance Change of Platinum Nanoparticles Assemblies. Biosens. Bioelectron..

[173] Stojko N.Y., Brainina K., Faller C., Henze G. (1998). Stripping Voltammetric Determination of Mercury at Modified Solid Electrodes. Anal. Chim. Acta.

[174] Liu W., Yao C., Cui H., Cang Y., Zhang Z., Miao Y., Xin Y. (2022). A Nano-Enzymatic Photoelectrochemical L-Cysteine Biosensor Based on Bi2MoO6 Modified Honeycomb TiO_2_ Nanotube Arrays Composite. Microchem. J..

[175] Ma J., Zhang M., Su W., Wu B., Yang Z., Wang X., Qiao B., Pei H., Tu J., Chen D. (2022). Photoelectrochemical Enzyme Biosensor Based on TiO_2_ Nanorod/TiO_2_ Quantum Dot/Polydopamine/Glucose Oxidase Composites with Strong Visible-Light Response. Langmuir.

[176] Yang Z., Xu W., Yan B., Wu B., Ma J., Wang X., Qiao B., Tu J., Pei H., Chen D. (2022). Gold and Platinum Nanoparticle-Functionalized TiO_2_ Nanotubes for Photoelectrochemical Glucose Sensing. ACS Omega.

[177] Kozitsina A.N., Svalova T.S., Malysheva N.N., Okhokhonin A.V., Vidrevich M.B., Brainina K.Z. (2018). Sensors Based on Bio and Biomimetic Receptors in Medical Diagnostic, Environment, and Food Analysis. Biosensors.

[178] Almeida E.S., Silva L.A.J., Sousa R.M.F., Richter E.M., Foster C.W., Banks C.E., Munoz R.A.A. (2016). Organic-Resistant Screen-Printed Graphitic Electrodes: Application to on-Site Monitoring of Liquid Fuels. Anal. Chim. Acta.

[179] Dzyadevych S.V., Arkhypova V.N., Soldatkin A.P., El’skaya A.V., Martelet C., Jaffrezic-Renault N. (2008). Amperometric Enzyme Biosensors: Past, Present and Future. IRBM.

[180] Chaubey A., Malhotra B.D. (2002). Mediated Biosensors. Biosens. Bioelectron..

[181] Wang K., Zhang R., Sun N., Li X., Wang J., Cao Y., Pei R. (2016). Near-Infrared Light-Driven Photoelectrochemical Aptasensor Based on the Upconversion Nanoparticles and TiO_2_/CdTe Heterostructure for Detection of Cancer Cells. ACS Appl. Mater. Interfaces.

[182] Mavrič T., Benčina M., Imani R., Junkar I., Valant M., Kralj-Iglič V., Iglič A. (2018). Electrochemical Biosensor Based on TiO_2_ Nanomaterials for Cancer Diagnostics. Adv. Biomembr. Lipid Self-Assem..

[183] Mostufa S., Akib T.B.A., Rana M.M., Islam M.R. (2022). Highly Sensitive TiO_2_/Au/Graphene Layer-Based Surface Plasmon Resonance Biosensor for Cancer Detection. Biosensors.

[184] Cui J., Wang X., Chen S. (2022). Ho_2_O_3_-TiO_2_ Nanobelts Electrode for Highly Selective and Sensitive Detection of Cancer MiRNAs. Biosensors.

[185] Khaliq N., Rasheed M.A., Khan M., Maqbool M., Ahmad M., Karim S., Nisar A., Schmuki P., Cho S.O., Ali G. (2021). Voltage-Switchable Biosensor with Gold Nanoparticles on TiO2 Nanotubes Decorated with CdS Quantum Dots for the Detection of Cholesterol and H_2_O_2_. ACS Appl. Mater. Interfaces.

[186] Eksin E., Erdem A. (2023). Recent Progress on Optical Biosensors Developed for Nucleic Acid Detection Related to Infectious Viral Diseases. Micromachines.

[187] Vadlamani B.S., Uppal T., Verma S.C., Misra M. (2020). Functionalized TiO_2_ Nanotube-Based Electrochemical Biosensor for Rapid Detection of SARS-CoV-2. Sensors.

[188] Sakib S., Pandey R., Soleymani L., Zhitomirsky I. (2020). Surface Modification of TiO_2_ for Photoelectrochemical DNA Biosensors. Med. Devices Sens..

[189] Yoo J., Jeong H., Park S.K., Park S., Lee J.S. (2021). Interdigitated Electrode Biosensor Based on Plasma-Deposited TiO_2_ Nanoparticles for Detecting DNA. Biosensors.

[190] Song K., Lin J., Zhuang Y., Han Z., Chen J. (2021). Construction of Photoelectrochemical DNA Biosensors Based on TiO_2_@Carbon Dots@Black Phosphorous Quantum Dots. Micromachines.

[191] Fan X., Sun N., Wang S., Xu M., Zuo C., Xu X., Li Z., Sun Q., Wang Y., Liu P. (2022). A Label-Free Electrochemiluminescence Sensing for Detection of Dopamine Based on TiO_2_ Electrospun Nanofibers. Electroanalysis.

[192] Wang Y., Yin L., Wu J., Li N., He N., Zhao H., Wu Q., Li X. (2021). Perovskite-SrTiO_3_/TiO_2_/PDA as Photoelectrochemical Glucose Biosensor. Ceram. Int..

[193] Sun Y., Li P., Zhu Y., Zhu X., Zhang Y., Liu M., Liu Y. (2021). In Situ Growth of TiO_2_ Nanowires on Ti_3_C_2_ MXenes Nanosheets as Highly Sensitive Luminol Electrochemiluminescent Nanoplatform for Glucose Detection in Fruits, Sweat and Serum Samples. Biosens. Bioelectron..

[194] Kadian S., Arya B.D., Kumar S., Sharma S.N., Chauhan R.P., Srivastava A., Chandra P., Singh S.P. (2018). Synthesis and Application of PHT-TiO_2_ Nanohybrid for Amperometric Glucose Detection in Human Saliva Sample. Electroanalysis.

[195] Ognjanović M., Stanković V., Knežević S., Antić B., Vranješ-Djurić S., Stanković D.M. (2020). TiO_2_/APTES Cross-Linked to Carboxylic Graphene Based Impedimetric Glucose Biosensor. Microchem. J..

[196] Kumar N., Chauhan N.S., Mittal A., Sharma S. (2018). TiO_2_ and Its Composites as Promising Biomaterials: A Review. BioMetals.

[197] Farajpour N., Deivanayagam R., Phakatkar A., Narayanan S., Shahbazian-Yassar R., Shokuhfar T. (2020). A Novel Antimicrobial Electrochemical Glucose Biosensor Based on Silver–Prussian Blue-modified TiO_2_ Nanotube Arrays. Med. Devices Sens..

[198] Yang W., Xu W., Wang Y., Chen D., Wang X., Cao Y., Wu Q., Tu J., Zhen C. (2020). Photoelectrochemical Glucose Biosensor Based on the Heterogeneous Facets of Nanocrystalline TiO_2_/Au/Glucose Oxidase Films. ACS Appl. Nano Mater..

[199] Akhbari Varkani R., Rafiee-Pour H.A., Noormohammadi M. (2021). One Step Immobilization of Glucose Oxidase on TiO_2_ Nanotubes towards Glucose Biosensing. Microchem. J..

[200] Wu B., Cheng Z., Hou Y., Chen Q., Wang X., Qiao B., Chen D., Tu J. (2022). Engineering Exposed Vertical Nano- TiO_2_(001) Facets/BiOI Nanosheet Heterojunction Film for Constructing a Satisfactory PEC Glucose Oxidase Biosensor. RSC Adv..

[201] Hussain M., Khaliq N., Khan A.A., Khan M., Ali G., Maqbool M. (2021). Synthesis, Characterization and Electrochemical Analysis of TiO_2_ Nanostructures for Sensing L-Cysteine and Hydrogen Peroxide. Phys. E Low-Dimens. Syst. Nanostruct..

[202] Saeed A.A., Abbas M.N., El-Hawary W.F., Issa Y.M., Singh B. (2022). A Core–Shell Au@TiO_2_ and Multi-Walled Carbon Nanotube-Based Sensor for the Electroanalytical Determination of H_2_O_2_ in Human Blood Serum and Saliva. Biosensors.

[203] Çakıroğlu B., Demirci Y.C., Gökgöz E., Özacar M. (2019). A Photoelectrochemical Glucose and Lactose Biosensor Consisting of Gold Nanoparticles, MnO_2_ and g-C_3_N_4_ Decorated TiO_2_. Sens. Actuators B Chem..

[204] Gunatilake U.B., Garcia-Rey S., Ojeda E., Basabe-Desmonts L., Benito-Lopez F. (2021). TiO_2_ Nanotubes Alginate Hydrogel Scaffold for Rapid Sensing of Sweat Biomarkers: Lactate and Glucose. ACS Appl. Mater. Interfaces.

[205] Umar A., Haque M., Ansari S.G., Seo H.K., Ibrahim A.A., Alhamami M.A.M., Algadi H., Ansari Z.A. (2022). Label-Free Myoglobin Biosensor Based on Pure and Copper-Doped Titanium Dioxide Nanomaterials. Biosensors.

[206] Huang L., Liang Z., Zhang F., Luo H., Liang R., Han F., Wu Z., Han D., Shen J., Niu L. (2022). Upconversion NaYF_4_:Yb/Er-TiO_2_-Ti_3_C_2_ Heterostructure-Based Near-Infrared Light-Driven Photoelectrochemical Biosensor for Highly Sensitive and Selective d-Serine Detection. Anal. Chem..

[207] Jiang M., Chen J.S. (2019). A Lable-Free ECL Biosensor for the Detection of Uric Acid Based on Au NRs at TiO_2_ Nanocomposite. Int. J. Electrochem. Sci..

[208] Parthasarathy P., Vivekanandan S. (2020). Biocompatible TiO_2_-CeO_2_ Nano-Composite Synthesis, Characterization and Analysis on Electrochemical Performance for Uric Acid Determination. Ain Shams Eng. J..

[209] Ramalingam M., Jaisankar A., Cheng L., Krishnan S., Lan L., Hassan A., Sasmazel H.T., Kaji H., Deigner H.P., Pedraz J.L. (2023). Impact of Nanotechnology on Conventional and Artificial Intelligence-Based Biosensing Strategies for the Detection of Viruses. Discov. Nano.

[210] Hu Z., Rong J., Zhan Z., Yu X. (2019). Glucose Biosensor Based on Glucose Oxidase Immobilized on Multi-Vacancy TiO_2_ Nanotube Arrays. Int. J. Electrochem. Sci..

[211] Jafari S., Mahyad B., Hashemzadeh H., Janfaza S., Gholikhani T., Tayebi L. (2020). Biomedical Applications of TiO_2_ Nanostructures: Recent Advances. Int. J. Nanomed..

[212] Yasri S., Wiwanitkit V. (2022). Sustainable Materials and COVID-19 Detection Biosensor: A Brief Review. Sens. Int..

[213] Kanwar V.S., Sharma A., Rinku, Kanwar M., Srivastav A.L., Soni D.K. (2023). An Overview for Biomedical Waste Management during Pandemic like COVID-19. Int. J. Environ. Sci. Technol..

[214] Hashemzadeh H., Javadi H., Darvishi M.H. (2020). Study of Structural Stability and Formation Mechanisms in DSPC and DPSM Liposomes: A Coarse-Grained Molecular Dynamics Simulation. Sci. Rep..

[215] Li Q., Wang X., Lu X., Tian H., Jiang H., Lv G., Guo D., Wu C., Chen B. (2009). The Incorporation of Daunorubicin in Cancer Cells through the Use of Titanium Dioxide Whiskers. Biomaterials.

[216] Kim C., Kim S., Oh W., Choi M., Jang J. (2012). Efficient Intracellular Delivery of Camptothecin by Silica/Titania Hollow Nanoparticles. Chem. A Eur. J..

[217] Chen J.-L., Zhang H., Huang X.-Q., Wan H.-Y., Li J., Fan X.-X., Luo K.Q., Wang J., Zhu X.-M., Wang J. (2019). Antiangiogenesis-Combined Photothermal Therapy in the Second Near-Infrared Window at Laser Powers below the Skin Tolerance Threshold. Nano-Micro Lett..

[218] Mushtaq A., Hou Y., Tian C., Deng T., Xu C., Sun Z., Kong X., Zubair Iqbal M. (2021). Facile Synthesis of Mn Doped TiO_2_ Rhombic Nanocomposites for Enhanced T1-Magnetic Resonance Imaging and Photodynamic Therapy. Mater. Res. Bull..

[219] Fei Yin Z., Wu L., Gui Yang H., Hua Su Y. (2013). Recent Progress in Biomedical Applications of Titanium Dioxide. Phys. Chem. Chem. Phys..

[220] Lee J., Jeong J.-S., Kim S.Y., Park M.-K., Choi S.-D., Kim U.-J., Park K., Jeong E.J., Nam S.-Y., Yu W.-J. (2019). Titanium Dioxide Nanoparticles Oral Exposure to Pregnant Rats and Its Distribution. Part. Fibre Toxicol..

[221] Liu X., Sui B., Sun J. (2017). Size- and Shape-Dependent Effects of Titanium Dioxide Nanoparticles on the Permeabilization of the Blood–Brain Barrier. J. Mater. Chem. B.

[222] Abdulnasser Harfoush S., Hannig M., Le D.D., Heck S., Leitner M., Omlor A.J., Tavernaro I., Kraegeloh A., Kautenburger R., Kickelbick G. (2020). High-Dose Intranasal Application of Titanium Dioxide Nanoparticles Induces the Systemic Uptakes and Allergic Airway Inflammation in Asthmatic Mice. Respir. Res..

[223] Lim J.-O., Lee S.-J., Kim W.-I., Pak S.-W., Moon C., Shin I.-S., Heo J.-D., Ko J.-W., Kim J.-C. (2021). Titanium Dioxide Nanoparticles Exacerbate Allergic Airway Inflammation via TXNIP Upregulation in a Mouse Model of Asthma. Int. J. Mol. Sci..

[224] Cui F., Zhou Z., Zhou H.S. (2020). Review—Measurement and Analysis of Cancer Biomarkers Based on Electrochemical Biosensors. J. Electrochem. Soc..

[225] Liu P., Huo X., Tang Y., Xu J., Liu X., Wong D.K.Y. (2017). A TiO_2_ Nanosheet-g-C_3_N_4_ Composite Photoelectrochemical Enzyme Biosensor Excitable by Visible Irradiation. Anal. Chim. Acta.

[226] Mansoor A., Khurshid Z., Khan M.T., Mansoor E., Butt F.A., Jamal A., Palma P.J. (2022). Medical and Dental Applications of Titania Nanoparticles: An Overview. Nanomaterials.

[227] Azzawi Z.G.M., Hamad T.I., Kadhim S.A., Naji G.A.-H. (2018). Osseointegration Evaluation of Laser-Deposited Titanium Dioxide Nanoparticles on Commercially Pure Titanium Dental Implants. J. Mater. Sci. Mater. Med..

[228] Souza J.C.M., Sordi M.B., Kanazawa M., Ravindran S., Henriques B., Silva F.S., Aparicio C., Cooper L.F. (2019). Nano-Scale Modification of Titanium Implant Surfaces to Enhance Osseointegration. Acta Biomater..

[229] Chen W., Shen X., Hu Y., Xu K., Ran Q., Yu Y., Dai L., Yuan Z., Huang L., Shen T. (2017). Surface Functionalization of Titanium Implants with Chitosan-Catechol Conjugate for Suppression of ROS-Induced Cells Damage and Improvement of Osteogenesis. Biomaterials.

[230] Ueno T., Ikeda T., Tsukimura N., Ishijima M., Minamikawa H., Sugita Y., Yamada M., Wakabayashi N., Ogawa T. (2016). Novel Antioxidant Capability of Titanium Induced by UV Light Treatment. Biomaterials.

[231] Yu Y., Shen X., Luo Z., Hu Y., Li M., Ma P., Ran Q., Dai L., He Y., Cai K. (2018). Osteogenesis Potential of Different Titania Nanotubes in Oxidative Stress Microenvironment. Biomaterials.

[232] Yu W., Jiang X., Zhang F., Xu L. (2010). The Effect of Anatase TiO_2_ Nanotube Layers on MC_3_T_3_-E_1_ Preosteoblast Adhesion, Proliferation, and Differentiation. J. Biomed. Mater. Res. Part A.

